# Operating-Regime Evaluation of Byzantine-Resilient Multi-Agent Reinforcement Learning for Sensor-Networked Safe Formation Control

**DOI:** 10.3390/s26144408

**Published:** 2026-07-11

**Authors:** Fuliang Ma, Yuping Ma, Yuzhen Dang, Yujun Ma, Hongbin Ma

**Affiliations:** 1 School of Chemical Engineering, Qinghai University, Xining 810016, China; 2 China Mobile Qinghai Company, Xining 810001, China

**Keywords:** Byzantine-resilient MARL, sensor networks, safe formation control, trust estimation, multi-agent reinforcement learning, cyber-physical systems, adversarial robustness, control barrier functions

## Abstract

Byzantine-resilient multi-agent reinforcement learning (MARL) matters in networked cyber-physical systems, where corrupted sensor messages degrade formation accuracy and execution-time safety. This paper presents an evaluation and audit study: a multiplicity-corrected operating-regime protocol applied to RS-MARL, a representative trust-based safety pipeline. The aim is to identify supported, inconclusive, and detector-limited regimes rather than claim a universally superior new MARL algorithm. The evidence base contains a 3000-run core matrix over five methods, six attack families, five Byzantine ratios, and 20 seeds per cell; 580 benign-control and ablation runs; and a 2380-run review-audit extension covering A-CBF calibration, four-switch ablation, sensor impairment, and high-seed confirmation. Results are regime-specific. RS-MARL has lower mean safety violations than Safe-MAPPO in 19 of 30 attack-ratio cells, but no core contrast survives Holm correction. Detection is reliable under collusive, random, and stealthy attacks, but weak or undefined under constant, adaptive, and sign-flip attacks, which bound the current energy-based trust detector’s operating envelope. A-CBF margin retuning does not improve over the deployed setting after correction, while four-switch ablation identifies SET as independently necessary for collusive-attack detection. The results support a reproducible reporting template: matched baselines, sensitivity estimates, detection reliability, artefact audits, and explicit safety-performance trade-offs.

## 1. Introduction

Safety-critical multi-agent reinforcement learning must coordinate under adversarial information without turning robustness into excessive conservatism. This tension is acute in formation control, where agents depend on shared state information while also satisfying collision, obstacle, and connectivity constraints. A defence that suppresses suspicious information can reduce Byzantine influence, but it can also remove useful coordination signals. A safety filter can reduce immediate violations, but it may push the formation away from its target geometry. The field therefore lacks a systematic answer to *when* such trade-offs are worth accepting, because Byzantine-resilient MARL studies rarely report the performance cost of their defences alongside safety gains.

A second reporting problem compounds this design problem. Robustness studies commonly sweep multiple attack families, corruption ratios, metrics, and baselines, then summarise the cells in which a defence appears favourable. Without seed-matched comparisons and family-wise correction for the resulting multiplicity, a nominally significant cell can be weak evidence for general resilience. The absence of a benign-control block further makes it difficult to distinguish useful Byzantine mitigation from nominal-regime conservatism. We therefore treat correction-aware evaluation itself as a central object of study, while using RS-MARL as the concrete safety-critical pipeline on which to instantiate the audit. This positioning is deliberate: the manuscript is primarily an operating-regime evaluation and auditing paper, and the algorithmic pipeline is used to make the evaluation protocol concrete.

Multi-agent reinforcement learning (MARL) has demonstrated flexible cooperative control under partial observation [1,2,3,4,5,6], with formation control serving as a demanding test case [7]. Agents must track desired geometry while avoiding collisions, obstacles, and connectivity failures. A method that improves reward but increases unsafe interactions is insufficient for safety-critical use.

Byzantine robustness, resilience to agents that behave arbitrarily, has been studied in federated learning, distributed optimisation, and resilient consensus [8,9,10,11,12,13,14,15]. In MARL, corrupted information affects both training and execution: a biased interaction perturbs critic estimates, alters future trajectories, and can cause unsafe physical behaviour. Three families of defence are relevant. Robust aggregation reduces the effect of malicious updates [13,14,15,16], but does not enforce collision or connectivity constraints. Safe MARL and CBF-based methods reduce constraint violations [17,18,19,20,21,22], but often assume benign communication. Trust and anomaly-detection methods identify suspicious interactions [23,24,25,26,27], yet imperfect detection can remove useful information. Few evaluations jointly measure these mechanisms and their resulting trade-offs.

These observations motivate the following research question:

*Under Byzantine perturbations in sensor-networked formation control, when do trust-aware safety mechanisms reduce execution-time safety violations, and what costs do they impose on formation accuracy and return?*

The pipeline under study, RS-MARL, is a safety-oriented Byzantine-resilient MARL framework with three coupled components. The statistical-energy trust estimator (SET) produces soft reliability scores from sliding-window message-energy statistics. The trust-weighted consensus critic (TWCC) uses these scores to reduce the influence of suspicious interactions during centralised training. The Adaptive CBF-inspired safety filter (A-CBF) modifies nominal actions during execution, expanding safety margins around low-trust agents. We use this pipeline as a representative trust-based safety design for the evaluation protocol; the paper does not rely on claiming that each component is independently novel or independently effective in all regimes. In this sense, RS-MARL is a case-study pipeline rather than the sole source of novelty; the main scientific claim concerns how Byzantine-resilient MARL should be evaluated and bounded.

Critically, we do not assume these components improve every metric. Defensive mechanisms are conservative by design: trust down-weighting can remove useful information alongside malicious signals, and action filtering can move the policy away from formation-error-minimising trajectories. We therefore treat the safety-performance trade-off as a central outcome rather than as a secondary implementation detail.

We evaluate RS-MARL under six Byzantine attack families (constant, random, sign-flip, stealthy, adaptive, and collusive) across five Byzantine ratios and 20 random seeds per cell. The comparison includes MAPPO and three baselines spanning robust aggregation (MAPPO-Krum and MAPPO-CWMed) and safety filtering (Safe-MAPPO). The validated evidence now contains a complete 5×6×5×20=3000 matched core matrix, a 100-run benign-control rerun, a 480-run canonical component-ablation rerun, and a 2380-run review-audit extension, giving 5960 audited runs in total. All experiments share the same pipeline, and the supplemental blocks are treated as mechanism and external-validity audits rather than as evidence of universal method dominance. The attack set is intentionally heterogeneous: distribution-shifting attacks test detector-supported regimes, whereas constant, adaptive, and sign-flip attacks test whether the trust mechanism fails when RMS-energy statistics are less informative. Matched seeds, fixed baselines, and correction across comparisons prevent the analysis from being driven by selectively favourable cells.

The results define operating regimes rather than a single ranking. The main message is conservative: RS-MARL is useful in selected regimes, inconclusive in many corrected contrasts, and detector-limited under specific attacks. RS-MARL has numerically lower mean safety violations than Safe-MAPPO in 19 of 30 attack-ratio cells, while Holm correction across 360 core contrasts limits the admissible claim to regime-level, cell-wise evidence rather than family-wise statistical dominance. A sensitivity audit shows that 20 paired seeds per cell are insufficient to rule out small effects after correction, so non-significant results are treated as inconclusive rather than as evidence of equivalence.

Detection is strong under collusive, random, and stealthy attacks (attack-family mean F1 > 0.98) but remains weak or undefined under constant, adaptive, and sign-flip attacks (constant recall =0; adaptive F1 ≤ 0.13; sign-flip F1 ≤ 0.13 where defined). The benign-control rerun reveals nominal-regime cost descriptively rather than as a Holm-significant degradation: under no attack, the best nominal metrics are set by MAPPO and MAPPO-CWMed rather than by RS-MARL. The 2380-run review-audit extension further shows that A-CBF calibration is not resolved by a simple margin-gain scan: no tested $g_{m}$ value significantly dominates the deployed $g_{m}=0.55$ after Holm correction. The stronger four-switch ablation separates trust estimation, critic weighting, neighbour-feature trust weighting, and safety-margin trust modulation; only SET removal causes Holm-significant detection-F1 loss in collusive regimes, while safety, formation-error, and return effects remain statistically unresolved. Crucially, these findings become visible only when the evidence is reported with matched baselines, uncertainty estimates, detection reliability, reproducibility audits, multiplicity correction, and quantified trade-offs.

This paper makes four contributions:

- The primary contribution is a multiplicity-corrected evaluation protocol for Byzantine-resilience claims in safety-critical MARL, combining complete seed-matched method-attack-ratio comparisons, paired seed-level testing, Holm correction, benign-control auditing, component-ablation auditing, and explicit operating-regime summaries.
- An operating-regime characterisation of trust-based safety defences that separates detector-supported regimes (collusive, random, and stealthy attacks) from detector-limited regimes (constant, adaptive, and sign-flip attacks) and from the benign regime where defensive conservatism carries nominal cost.
- An audited evidence base comprising a 3000-run core matrix, 580 canonical supplemental runs, and a 2380-run review-audit extension, with matrix-completeness auditing, labelled source-data blocks, per-seed metrics, SHA256-verified artefacts, and analysis scripts for independent re-analysis.
- A representative case study, RS-MARL, integrating statistical-energy trust estimation, trust-weighted critic learning, and adaptive CBF-inspired execution-time filtering to demonstrate how the protocol distinguishes local cell-wise improvements from family-wise statistical support and module-level coupling. This case study is not used to claim a new optimisation or control primitive, but to make the evaluation framework auditable.

The remainder of this paper is organised as follows. Section 2 reviews related work. Section 3 formulates the Byzantine-resilient formation-control problem. Section 4 presents the RS-MARL case-study pipeline. Section 5 provides an analytical interpretation of the mechanism assumptions. Section 6 reports the experimental results. Section 7 discusses limitations and implications. Section 8 concludes the paper.

## 2. Related Work

### 2.1. Byzantine Robustness in Distributed and Multi-Agent Learning

Byzantine robustness has been widely studied in distributed optimisation, federated learning, and networked multi-agent systems, where a subset of workers or agents may behave arbitrarily and corrupt gradients, model updates, or communicated information [13,14,15,16,28]. Recent work has also considered Byzantine robustness in cooperative MARL, multi-UAV coordination, decentralised policy evaluation, and federated policy-gradient settings [8,9,10,11]. In resilient consensus and formation tracking, adversarial agents can disrupt collective behaviour, motivating secure coordination and resilient output-tracking mechanisms [12,29,30,31].

However, Byzantine robustness in MARL introduces additional challenges. Compromised agents may affect not only parameter updates but also observations, messages, actions, rewards, or critic inputs. Moreover, their influence propagates through sequential decision-making and agent interactions. A corrupted signal at one time step can alter the future state distribution and induce unsafe behaviours. Recent Byzantine-resilient MARL studies have considered UAV coordination and decentralised policy evaluation, but they do not jointly address trust estimation, critic weighting, and execution-time safety filtering. Robust aggregation methods are useful baselines, but they do not directly address physical safety constraints during execution. RS-MARL differs from aggregation-only approaches by coupling trust-aware learning with execution-time safety filtering.

### 2.2. Safe MARL and Control Barrier Functions

Safe reinforcement learning aims to optimise task performance while satisfying constraints, and safety validation for autonomous systems has become a prominent application of reinforcement-learning methods [32]. In multi-agent settings, safety requirements often include inter-agent collision avoidance, obstacle avoidance, connectivity maintenance, communication constraints, and bounded control inputs. Recent safe MARL and safe multi-robot control studies have considered constrained policy optimisation, safe cooperative navigation, scalable constrained learning, and verification-oriented safety design [17,18,20,33]. Control barrier functions provide a principled tool for enforcing state-dependent safety constraints, and recent neural and graph-based CBF methods extend safety filtering to distributed multi-agent control [19,21,22,34,35].

These methods can reduce safety violations in cooperative control tasks. However, many safe MARL approaches assume benign agents or stochastic disturbances rather than strategic Byzantine adversaries. When agents exchange corrupted information, a safety filter may still help reduce violations, but it may also become conservative because the nominal policy and estimated local state may be affected by adversarial perturbations. RS-MARL incorporates an adaptive CBF-inspired safety filter and couples it with trust-aware Byzantine mitigation.

### 2.3. Trust Estimation and Anomaly Detection

Trust estimation and anomaly detection are important tools for resilient multi-agent systems. In adversarial or uncertain environments, agents may assign reliability scores to neighbours based on behavioural consistency, message reliability, prediction errors, communication patterns, or statistical deviations [23,24,25]. Related work on secure state estimation and cyber-physical attack detection studies how to infer and mitigate corrupted measurements or false-data injection attacks [27,36,37]. Graph anomaly detection and spectral deviation methods provide additional tools for identifying structured deviations in interaction data [26,38,39,40].

In MARL, trust mechanisms can reduce the influence of non-cooperative or malicious agents on value estimation, policy updates, or message passing. However, trust estimation is challenging because benign agents may also deviate from expected behaviour due to exploration, partial observability, environmental disturbances, or transient formation errors. As a result, trust estimators face a precision–recall trade-off. RS-MARL uses a statistical-energy trust estimator that detects suspicious interactions from consistency and message-energy signals and incorporates trust scores into critic learning and safety filtering.

### 2.4. Safety-Performance Trade-Offs in Robust MARL

Robustness and safety mechanisms often introduce performance trade-offs. Robust aggregation can reduce the impact of corrupted updates but does not directly enforce safe execution [13,15]. Conversely, CBF-based filters can reduce constraint violations but may move actions away from task-optimal controls [19,22,34]. In robust or adversarial reinforcement learning, defensive mechanisms may improve worst-case behaviour while degrading nominal performance [41,42,43]. In MARL, these effects are amplified because one agent’s conservative action may affect team-level coordination. This motivates evaluating Byzantine-resilient MARL not only by return or formation-error performance, but also by safety violations, detection reliability, and the trade-offs induced by defensive mechanisms.

### 2.5. Evaluation Practice in Robust and Byzantine MARL

Beyond the design of defences, the reporting protocol determines what can be concluded from a robustness study. Byzantine-resilient and adversarial MARL evaluations often sweep multiple attack families, corruption ratios, baselines, and metrics [8,9,10,11,41,42]. Such sweeps are necessary, but they also create many opportunities for favourable cell-wise comparisons. If each cell is interpreted independently, a method can appear broadly robust even when the effect does not survive family-wise correction. Similarly, evaluations that omit a no-attack control cannot determine whether a defence improves robustness at an acceptable nominal-regime cost.

This paper targets that reporting gap directly. Rather than relying on a single aggregate score or selected favourable cells, we use a complete matched matrix, paired seed-level tests, Holm correction, a benign-control rerun, component-ablation auditing, and an explicit operating-regime summary. The goal is not to dismiss existing defence mechanisms, but to make their evidence admissible: a method should state where it helps, where it is not yet supported, where detection is unreliable, and where safety gains are purchased by degraded nominal coordination.

## 3. Materialsand Methods

### 3.1. Multi-Agent Formation Control

We consider a cooperative formation-control problem with *N* agents:

(1) N={1,…,N}.

Each agent i∈N has state $x_{i}^{t}\in X_{i}$, observation $o_{i}^{t}\in O_{i}$, and action $a_{i}^{t}\in A_{i}$ at time step *t*. The joint state and joint action are denoted by

(2) $$x^{t}=\left(x_{1}^{t},\ldots ,x_{N}^{t}\right),\;a^{t}=\left(a_{1}^{t},\ldots ,a_{N}^{t}\right).$$

Agents learn decentralised policies

(3) $$\pi_{i}\left(a_{i}^{t}|o_{i}^{t}\right),\;i\in N,$$

to maximise the expected discounted return:

(4) $$J\left(\pi \right)=E_{\pi ,P}\left[\sum_{t=0}^{T-1}\gamma^{t}r\left(x^{t},a^{t}\right)\right].$$

Let $p_{i}^{t}\in R^{d}$ denote the position of agent *i*, and let $d_{ij}^{⋆}$ denote the desired relative distance between agents *i* and *j*. A representative formation error is

(5) $$e_{\mathrm{form}}^{t}=\frac{1}{\left|E\right|}\sum_{(i,j)\in E}\left|∥p_{i}^{t}-p_{j}^{t}∥-d_{ij}^{⋆}\right|,$$

where E is the set of formation edges.

### 3.2. Sensor-Networked Observation and Communication Model

We model the formation-control system as a network of mobile sensor nodes. Each agent uses local sensing and localisation to estimate its own kinematic state and receives state reports from neighbouring agents through the communication graph. In the simulator, a message from sender *j* to receiver *i* is represented as a control-relevant sensor-network report $m_{ij}^{t}$ containing the sender’s kinematic information and formation-relevant quantities used to construct neighbour summaries. The receiver then forms its observation from local position, velocity, target displacement, and aggregated neighbour features. This abstraction captures the setting in which compromised nodes falsify communicated sensor-derived state reports rather than directly modifying the receiver’s physical state.

The communication graph is fixed by a sensing/communication radius, and all core methods are evaluated under the same graph, sampling horizon, and observation dimension. Byzantine attacks act on the received reports ${\tilde{m}}_{ij}^{t}$, so the adversarial surface is the sensor-network communication channel. The 3000-run core matrix isolates falsified sensor-report attacks without packet loss, asynchronous delays, heterogeneous sensor modalities, or measurement-noise sweeps. The review-audit V2 extension adds a bounded sensor-impairment mini-grid with packet loss, observation noise, and one-step message delay to test whether the operating-regime conclusions are stable under modest communication and sensing degradation. Because this mini-grid covers only two methods and selected domains, it is interpreted as an external-validity audit rather than as a full sensor-network robustness claim.

### 3.3. Byzantine Agent Model

A subset of agents may be Byzantine. Let

(6) B⊆N

denote the set of Byzantine agents, and let

(7) $$\rho =\frac{\left|B\right|}{N}$$

be the Byzantine ratio. Byzantine agents may corrupt observations, messages, or control-relevant information. Unlike stochastic disturbances, Byzantine perturbations can be strategic, time-varying, and coordinated.

Let $m_{ij}^{t}$ denote the information sent from agent *j* to agent *i*. For a Byzantine agent j∈B, the received message may be corrupted as

(8) $${\tilde{m}}_{ij}^{t}=A_{j}^{t}\left(m_{ij}^{t},x^{t},a^{t},\xi^{t}\right),$$

where $A_{j}^{t}$ is an attack function and $\xi^{t}$ denotes attack randomness or adversarial side information. We consider six attack types: constant, random, sign-flip, stealthy, adaptive, and collusive attacks.

### 3.4. Safety Constraints

The formation-control task is safety-critical. We consider collision avoidance, obstacle avoidance, and connectivity maintenance. For collision avoidance,

(9) $$h_{ij}^{\mathrm{col}}\left(x^{t}\right)={∥p_{i}^{t}-p_{j}^{t}∥}^{2}-d_{\min}^{2}\geq 0.$$

For obstacle avoidance,

(10) $$h_{io}^{\mathrm{obs}}\left(x^{t}\right)={∥p_{i}^{t}-c_{o}∥}^{2}-r_{o}^{2}\geq 0.$$

For connectivity maintenance,

(11) $$h_{ij}^{\mathrm{con}}\left(x^{t}\right)=d_{\max}^{2}-{∥p_{i}^{t}-p_{j}^{t}∥}^{2}\geq 0.$$

The overall safe set is

(12) $$C=\left\{x:h_{k}\left(x\right)\geq 0,\;\forall k\in K\right\}.$$

### 3.5. Trust and Detection Metrics

RS-MARL assigns trust scores to interactions. Let

(13) $$\tau_{ij}^{t}\in \left[0,1\right]$$

denote the trust score assigned by agent *i* to information from agent *j*. A high value indicates reliability, while a low value indicates suspicious behaviour. Given a threshold η, an interaction may be classified as suspicious if $\tau_{ij}^{t}<\eta$.

For evaluation, Byzantine detection is measured using precision, recall, and F1:

(14) $$\mathrm{Precision}=\frac{\mathrm{TP}}{\mathrm{TP}+\mathrm{FP}},$$

(15) $$\mathrm{Recall}=\frac{\mathrm{TP}}{\mathrm{TP}+\mathrm{FN}},$$

(16) $$F1=\frac{2\cdot \mathrm{Precision}\cdot \mathrm{Recall}}{\mathrm{Precision}+\mathrm{Recall}}.$$

### 3.6. Evaluation Objective

The objective is to learn decentralised policies and trust-aware safety mechanisms that jointly address task performance, Byzantine robustness, and safety. We evaluate methods using formation error, task return, safety violations, and detection precision, recall, and F1. This multi-metric formulation is necessary because a defence may reduce safety violations while increasing formation error or reducing return.

## 4. Case-Study Pipeline: RS-MARL

### 4.1. Overview

RS-MARL is a safety-oriented Byzantine-resilient MARL framework for formation control. It consists of three coupled components: a statistical-energy trust estimator (SET), a trust-weighted consensus critic (TWCC), and an adaptive CBF-inspired safety filter (A-CBF). SET and TWCC address corrupted information during centralised training, while A-CBF provides safety-aware action correction during decentralised execution. The modules are coupled through interaction-level trust scores: trust influences critic aggregation during training and modulates the conservatism of the safety filter during execution.

Importantly, RS-MARL is not designed to improve every metric. Trust down-weighting and safety filtering can introduce conservative behaviour. Therefore, RS-MARL should be understood as a safety-oriented robust MARL framework rather than a universally performance-improving controller. This framing also limits the algorithmic claim: the pipeline combines known trust, aggregation, and safety-filtering ideas, while the evaluation protocol tests when such a combination is supported by corrected evidence.

### 4.2. Statistical-Energy Trust Estimator

Let ${\tilde{m}}_{j}^{t}\in R^{d_{m}}$ denote the message vector broadcast by sender *j* at time *t*. In the released implementation, this vector contains the sender state components used by the formation controller. Each receiver maintains a sliding window $W_{j}^{t}={\left\{{\tilde{m}}_{j}^{t-\ell }\right\}}_{\ell =0}^{W-1}$ for every sender and computes a sender-energy statistic

(17) $$e_{j}^{t}={\left(\frac{1}{|W_{j}^{t}|d_{m}}\sum_{\tilde{m}\in W_{j}^{t}}{∥\tilde{m}-{\bar{m}}_{j}^{t}∥}_{2}^{2}\right)}^{1/2},$$

where ${\bar{m}}_{j}^{t}$ is the window mean. This statistic measures short-horizon message variability and is inexpensive to update online.

For receiver *i*, the energy values of its valid neighbours are normalised by robust median and MAD statistics. Let $q_{ij}^{t}={\left[e_{j}^{t},e_{j}^{t}\right]}^{⊤}$ denote the two-channel energy feature used in the implementation. The two entries are intentionally identical: the duplication preserves the fixed two-channel trust-feature interface used by the released runner and should not be interpreted as two independent statistics. The normalised anomaly score is

(18) $$z_{ij}^{t}=\sum_{\ell =1}^{2}\left|\frac{q_{ij,\ell }^{t}-{\mathrm{median}}_{k\in N_{i}}\left(q_{ik,\ell }^{t}\right)}{{\mathrm{MAD}}_{k\in N_{i}}\left(q_{ik,\ell }^{t}\right)+\epsilon }\right|.$$

The instantaneous reliability is then

(19) $${\hat{\tau }}_{ij}^{t}=\exp\left(-z_{ij}^{t}/T\right),$$

where T>0 is a temperature. The trust score is updated through exponential smoothing:

(20) $$\tau_{ij}^{t+1}=\left(1-\lambda \right)\tau_{ij}^{t}+\lambda {\hat{\tau }}_{ij}^{t}.$$

Larger robust energy deviations therefore produce lower reliability, while the moving average prevents single-step transients from immediately dominating downstream control. These trust scores are soft reliability weights rather than exact Byzantine labels; false positives and false negatives are expected when Byzantine deviations overlap with nominal formation transients.

### 4.3. Trust-Weighted Consensus Critic

Let $Q_{\phi }\left(x^{t},a^{t};\tau^{t}\right)$ denote the centralised critic. Instead of aggregating all neighbour features uniformly, TWCC computes trust-weighted local features:

(21) $$z_{i}^{t}=\sum_{j\in N_{i}}w_{ij}^{t}f_{j}^{t},$$

where $f_{j}^{t}$ is the feature representation associated with neighbour *j*, and

(22) $$w_{ij}^{t}=\frac{\tau_{ij}^{t}}{\sum_{k\in N_{i}}\tau_{ik}^{t}+\epsilon }.$$

The direction of trust is important: $\tau_{ij}^{t}$ is the reliability assigned by receiver *i* to information originating from sender *j*. Thus, a Byzantine sender contributes to agent *i*’s critic input only through the total weight assigned by its receivers.

The critic is trained by minimising the temporal-difference loss

(23) $$L_{Q}\left(\phi \right)=E\left[{\left(Q_{\phi }\left(x^{t},a^{t};\tau^{t}\right)-y^{t}\right)}^{2}\right],$$

where

(24) $$y^{t}=r^{t}+\gamma Q_{\bar{\phi }}\left(x^{t+1},a^{t+1};\tau^{t+1}\right).$$

TWCC mitigates detectable or partially detectable adversarial behaviour by reducing the critic influence of low-trust interactions. It does not assume that all Byzantine influence can be removed, and its effectiveness depends on the separation between benign and adversarial residual patterns. This information pathway is also important for interpreting the ablation results: in the released runner, trust scores can affect the neighbour-feature summary, the critic-weighting path, and the trust-modulated safety margin. The canonical no-TWCC ablation did not isolate critic-loss weighting cleanly, so the review-audit V2 extension adds separate switches for critic-loss weighting, neighbour-feature trust weighting, and safety-margin trust modulation. We report module effects only where those stronger switches provide separable evidence.

### 4.4. Adaptive CBF-Inspired Safety Filter

Given a nominal action $a_{i,\mathrm{nom}}^{t}$ produced by the learned policy, the released safety module applies a lightweight CBF-inspired correction. Under the deployed parameterisation, the trust-adaptive margin gain is $g_{m}=0.55$ relative to $d_{\mathrm{safe}}=0.75$, so low-trust neighbours can substantially expand the repulsion margin. The filter should therefore be interpreted as a CBF-inspired safety layer with trust-modulated geometric margins, not as a hard CBF-QP certificate. For each pair of agents, the trust-adaptive margin is

(25) $$m_{ij}^{t}=d_{\mathrm{safe}}+g_{m}\left(1-\tau_{ij}^{t}\right),$$

where $d_{\mathrm{safe}}$ is the nominal collision distance and $g_{m}$ is the adaptive margin gain. If $d_{ij}^{t}={∥p_{i}^{t}-p_{j}^{t}∥}_{2}$, the pairwise repulsion term is

(26) $$r_{i,\mathrm{aa}}^{t}=\sum_{j\neq i}\left[\frac{{\left(m_{ij}^{t}-d_{ij}^{t}\right)}_{+}}{m_{ij}^{t}+\epsilon }\right]\left(p_{i}^{t}-p_{j}^{t}\right),$$

where ${\left(x\right)}_{+}=\max\left\{x,0\right\}$. For obstacle centre $c_{o}$ and obstacle radius $r_{o}$, the obstacle-boundary distance is $d_{io}^{t}={∥p_{i}^{t}-c_{o}∥}_{2}-r_{o}$, and

(27) $$r_{i,\mathrm{obs}}^{t}=\sum_{o}\left[\frac{{\left(d_{\mathrm{safe}}-d_{io}^{t}\right)}_{+}}{d_{\mathrm{safe}}+\epsilon }\right]\frac{p_{i}^{t}-c_{o}}{{∥p_{i}^{t}-c_{o}∥}_{2}+\epsilon }.$$

The executed action is

(28) $$a_{i,\mathrm{safe}}^{t}=\Pi_{∥a∥\leq a_{\max}}\left(a_{i,\mathrm{nom}}^{t}+k_{r}\left(r_{i,\mathrm{aa}}^{t}+r_{i,\mathrm{obs}}^{t}\right)\right),$$

where Π clips the acceleration norm to the actuation bound. This implementation avoids solving an online quadratic program, which keeps the safety layer inexpensive, but it should be interpreted as a CBF-inspired safety filter rather than a certificate of hard forward invariance. The filter becomes more conservative near safety boundaries or when local information is assigned low trust, at the cost of moving the executed action away from the formation-tracking action.

### 4.5. Integrated Training and Execution

Algorithm 1 summarises the training procedure. At each step, agents update interaction-level trust scores from normalised residuals and windowed energy deviations, execute the A-CBF-corrected action, and store the transition for critic and policy updates. During evaluation, the learned decentralised policies and the A-CBF filter are executed online using the same trust update rule; the centralised critic is used only during training.
**Algorithm 1** RS-MARL Training1:  Initialise policies ${\left\{\pi_{\theta_{i}}\right\}}_{i=1}^{N}$, critic $Q_{\phi }$, trust scores $\tau_{ij}=1$, and replay buffer D.2:  **for** episode =1,…,M **do**3:     Reset environment and initialize agent states.4:     **for** t=0,…,T−1 **do**5:       **for** each agent *i* **do**6:          Receive observation $o_{i}^{t}$ and neighbour information ${\tilde{m}}_{ij}^{t}$.7:          Update the sliding message-energy statistic and robust anomaly score $z_{ij}^{t}$.8:          Update trust score $\tau_{ij}^{t}$ using the exponential moving average.9:          Sample nominal action $a_{i,\mathrm{nom}}^{t}∼\pi_{\theta_{i}}\left(\cdot |o_{i}^{t}\right)$.10:        Apply A-CBF to obtain $a_{i,\mathrm{safe}}^{t}$.11:     **end for**12:     Execute safe joint action $a_{\mathrm{safe}}^{t}$ and store the transition in D.13:   **end for**14:   Update trust-weighted critic $Q_{\phi }$ and then update policies using advantage estimates from $Q_{\phi }$.15:**end for**

## 5. Analytical Interpretation

The analysis below formalises the conditions under which the three components of RS-MARL can mitigate Byzantine influence. We stress that these statements are *conditional*: they identify what must hold for the mechanism to work, rather than claiming those conditions are always satisfied or that the implemented filter provides a hard safety certificate. The experimental results in Section 6 independently test whether these conditions hold in practice.

**Assumption** **1** (Bounded signals)**.**
*States, actions, rewards, and neighbour features are bounded: $\left|r\left(x,a\right)\right|\leq R_{\max}$, $∥a∥\leq A_{\max}$, $∥f_{j}^{t}∥\;\leq F_{\max}$.*

**Assumption** **2** (Lipschitz value and dynamics)**.**
*The critic and one-step formation-error dynamics are Lipschitz continuous with constants $L_{Q}$ and $L_{e}$.*

**Assumption** **3** (Detectable Byzantine residual separation)**.**
*For detectable Byzantine interactions, there exists $\Delta_{s}>0$ such that the expected anomaly score for Byzantine senders exceeds that for benign senders by at least $\Delta_{s}$. This assumption is attack-dependent: it is empirically supported under collusive, random, and stealthy attacks (F1 > 0.98; Section 6.8) and empirically violated under constant, adaptive, and sign-flip attacks (constant recall =0; adaptive F1 ≤ 0.13; sign-flip F1 ≤ 0.13 where defined).*

**Proposition** **1** (Trust-weighted critic perturbation bound)**.**
*Let $z^{t}$ denote the ideal benign aggregate feature and ${\hat{z}}^{t}$ the trust-weighted feature used by TWCC. Under Assumptions 1 and 2 and a bounded trust-estimation error $\varepsilon_{\tau }$, the feature perturbation is bounded by*

(29) $$∥{\hat{z}}^{t}-z^{t}∥\;\leq C_{1}\varepsilon_{\tau }F_{\max}+C_{2}{\bar{w}}_{B}\Delta_{B},$$

*where ${\bar{w}}_{B}$ is the total receiver-side trust weight assigned to Byzantine senders and $\Delta_{B}$ bounds Byzantine feature deviation. Consequently, $|Q_{\phi }\left({\hat{z}}^{t},a^{t}\right)-Q_{\phi }\left(z^{t},a^{t}\right)|\;\leq L_{Q}\left(C_{1}\varepsilon_{\tau }F_{\max}+C_{2}{\bar{w}}_{B}\Delta_{B}\right)$. The bound isolates two limiting terms: imperfect trust estimation ($\varepsilon_{\tau }>0$) allows Byzantine features to enter the critic, and residual Byzantine weight (${\bar{w}}_{B}>0$) directly perturbs the aggregate. Both terms can remain non-zero in practice, consistent with reduced detection reliability under constant, adaptive, and sign-flip attacks.*

**Remark** **1** (Scope of the theoretical statements)**.**
*The above statements formalise sufficient conditions for Byzantine mitigation, not guaranteed outcomes. Assumption 3 is the key empirical gate: when detection fails, the perturbation bound is loose and the trust-weighted critic offers no formal advantage over uniform aggregation. The CBF-inspired safety filter provides a repulsive correction whose magnitude is bounded by the action clipping constant $A_{\max}$, and the deployed trust-adaptive margin gain ($g_{m}=0.55$ relative to base safe distance 0.75) can make the correction conservative when trust scores are low or noisy. The safety-performance trade-off emerges from two sources that the perturbation bound makes explicit: trust down-weighting reduces ${\hat{z}}^{t}$ relative to $z^{t}$ (sacrificing coordination information), and safety filtering introduces a non-zero action deviation $\Delta a^{t}$ that can increase formation error by up to $L_{e}∥\Delta a^{t}∥$.*

## 6. Results

### 6.1. Audited Experimental Matrix

We evaluate RS-MARL on a multi-agent formation-control benchmark under Byzantine perturbations. The task requires agents to maintain a desired formation while satisfying safety-related constraints. The core matched comparison includes MAPPO, Safe-MAPPO, MAPPO-Krum, MAPPO-CWMed, and RS-MARL over six attack models, five Byzantine ratios, and 20 random seeds per cell. The validated core dataset contains a complete 5×6×5×20=3000 per-seed matrix. The V3 publication data package adds two canonical same-pipeline supplemental blocks: a 100-run benign-control rerun for the no-attack nominal regime and a 480-run component-ablation rerun for adaptive, collusive, and constant attacks at Byzantine ratios 0.10 and 0.30. The review-audit V2 return package adds 2380 completed runs: a 700-run A-CBF margin-gain calibration scan, a 600-run four-switch ablation, a 480-run sensor-impairment mini-grid, and a 600-run n=100 key-domain confirmation block. We use the core matrix for baseline claims and the supplemental blocks to audit nominal cost, calibration sensitivity, module coupling, sensor-impairment robustness, and power-sensitive key regimes. Table 1 summarises the resulting validated matrix and evidence blocks.

This design is intended to answer an operating-regime question rather than to produce a single leaderboard. The core matrix tests method-attack-ratio behaviour under matched seeds; the benign-control block checks whether robustness is purchased by nominal-regime cost; the ablation blocks test whether the trust and safety modules can be interpreted separately; and the review-audit extensions check calibration, sensor impairment, and higher-seed uncertainty in targeted domains. Table 2, Table 3 and Table 4 report the runner constants that define this protocol.

**Environment details.** The benchmark contains N=30 agents operating in a two-dimensional workspace. Each agent observes its local position, velocity, target displacement, and trust-weighted neighbour summaries, resulting in a 10-dimensional observation. Each agent outputs a 2-dimensional continuous acceleration. The communication graph is induced by a fixed communication radius, and the desired formation is a lattice-like distance-based formation defined by the edge set E and desired pairwise distances ${\left\{d_{ij}^{⋆}\right\}}_{(i,j)\in E}$.

The reward consists of formation-error performance, safety, and control-effort terms:

(30) $$r^{t}=-w_{f}e_{\mathrm{form}}^{t}-w_{s}v_{\mathrm{safe}}^{t}-w_{u}{∥a^{t}∥}^{2},$$

where $e_{\mathrm{form}}^{t}$ is the formation error and $v_{\mathrm{safe}}^{t}$ is the time-step-level count of active safety-constraint violation events, aggregated over collision, obstacle, and connectivity constraints. The reported final_safety_violations metric uses the same event-count definition averaged over the final evaluation episodes for each seed. The constants $w_{f},w_{s},w_{u}$ are task weights. All methods use the same reward function, dynamics, episode horizon, attack configuration, and evaluation protocol.

**Attack implementation.** We evaluate six Byzantine attack models. In the constant attack, Byzantine agents inject a fixed bias into communicated or control-relevant information. In the random attack, Byzantine agents add random perturbations sampled from a bounded distribution. In the sign-flip attack, selected messages or control-relevant signals are multiplied by a negative factor. In the stealthy attack, perturbations are constrained to remain close to nominal interaction statistics while still degrading coordination. In the attack labelled adaptive in the released runner, Byzantine agents use a target-relative direction to move communicated positions away from the formation target; we therefore interpret it as a state-dependent target-deviation attack rather than as a fully strategic online adversary. In the collusive attack, multiple Byzantine agents coordinate their perturbations to produce correlated misleading signals. The Byzantine ratio ρ determines the fraction of compromised agents, and the same attack configuration is used across methods for fair comparison. Table 3 lists the released perturbation rules. Constant, adaptive, and sign-flip families are especially important for scope assessment because they can mimic or preserve parts of benign RMS-energy statistics, making them difficult for the current energy-based trust estimator.

### 6.2. Evaluation Metrics

We report formation error, task return, safety violations, and detection precision, recall, and F1. Lower formation error and safety violations are better, while higher return is better. Safety violations count violations of collision, obstacle, and connectivity-related constraints. Unless otherwise stated, final metrics are averaged over the five final evaluation episodes for each seed and then averaged over 20 random seeds.

### 6.3. Statistical Protocol and Matrix Completeness

For seed-level comparisons, we use paired Wilcoxon signed-rank tests when the same seed is available for RS-MARL and the comparison method under the same attack-ratio setting. If matched seeds are unavailable but both methods have at least two valid runs, the analysis script falls back to an unpaired Mann–Whitney *U* test and marks the comparison accordingly. In the validated core dataset, every method-attack-ratio cell contains the same 20 seeds; therefore, all 360 core tests are paired comparisons. We apply Holm correction within each metric family for metric-specific inference and additionally report a stricter global Holm audit across all 360 contrasts. The canonical ablation block is paired by seed and produces 54 component-comparison tests; these are interpreted descriptively because no adjusted comparison is significant. The review-audit V2 tests are likewise interpreted by audit family: A-CBF calibration is compared against the deployed $g_{m}=0.55$, four-switch ablations are compared against the full pipeline, the sensor-impairment mini-grid compares RS-MARL and Safe-MAPPO within selected impairment domains, and the n=100 block retests three key domains with substantially higher seed count. Table 5 confirms that the core matched-comparison matrix is complete, and Table 6 separates the evidence blocks used for claims.

We additionally report a sensitivity audit for the paired seed-level tests. With n=20 paired seeds, an approximate two-sided paired-test calculation gives a minimum detectable standardised effect of d≈0.63 at 80% power under uncorrected α=0.05, and d≈1.04 under a Bonferroni-scale proxy for the 360-contrast global family. At n=100, the corresponding detectable effects decrease to d≈0.28 and d≈0.47, respectively. These calculations are not substitutes for the non-parametric tests used in the main analysis, but they clarify interpretation: failure to survive Holm correction at n=20 should be read as insufficient evidence for a family-wise claim, not as evidence that the methods are equivalent or that small effects are absent.

### 6.4. Fresh Benign-Control and Component-Ablation Evidence

The canonical V3 supplemental data change the interpretation of the earlier draft in two ways. First, the benign-control rerun quantifies the nominal cost of the defence descriptively. Under no Byzantine attack, RS-MARL does not improve the nominal operating regime: MAPPO-CWMed gives the lowest mean safety-violation count, MAPPO gives the lowest mean formation error, and both MAPPO and MAPPO-CWMed obtain higher mean returns than RS-MARL (Table 7). These benign differences are not used to claim Holm-significant degradation, and no equivalence test with a pre-specified margin was registered. The correct interpretation is therefore narrower: the benign block prevents the robustness argument from being overstated and shows that RS-MARL should not be treated as a default nominal controller when Byzantine risk is absent.

Second, the canonical component-ablation rerun provides a same-pipeline audit of module-level trade-offs but does not yield module-wise statistical dominance. A configuration audit identified that the original use_trust_weighted_critic pathway was not independently identified by the 480-run canonical ablation block: the no-SET and no-TWCC variants had identical summary values and identical per-seed final metrics in all six tested attack-ratio cells. This block is therefore retained as a diagnostic audit rather than as evidence for a critic-only contribution. It also raised a calibration concern for the deployed safety filter, because removing the original A-CBF filter improved formation error and return in all six canonical ablation cells and lowered safety violations in five of six cells (Table 8). No canonical ablation comparison survives Holm correction across the 54 component tests, so these results motivate the stronger review-audit extension rather than supporting definitive module attribution.

### 6.5. Review-Audit Extension: Calibration, Four-Switch Ablation, Sensor Impairment, and High-Seed Confirmation

The 2380-run review-audit V2 extension was designed to address the main residual threats raised by the canonical evidence block. First, the A-CBF calibration scan tests adaptive margin gains $g_{m}\in \left\{0,0.10,0.20,0.35,0.55\right\}$ over benign, adaptive, collusive, and sign-flip domains. Averaged across the seven audited cells, the deployed $g_{m}=0.55$ gives the lowest mean safety-violation count, lowest mean formation error, and highest mean return (Table 9). However, no comparison against $g_{m}=0.55$ survives Holm correction (minimum raw p=0.0144, all adjusted p=1.000). Thus, the scan does not prove that the deployed setting is optimal, but it does rule out the stronger claim that a simple tested margin value clearly dominates it.

Second, the four-switch ablation separates trust estimation, critic-loss weighting, neighbour-feature trust weighting, and trust-dependent safety-margin modulation. This resolves the earlier no-SET/no-TWCC ambiguity. The strongest corrected effect is specific to detection: disabling SET substantially reduces detection F1 in collusive regimes, and the SET-disabled detection-F1 losses remain significant after Holm correction. In contrast, no safety, formation-error, or return comparison is significant after correction (Table 10). The appropriate interpretation is that SET is independently necessary for collusive-attack detection, whereas independent performance or safety contributions of the downstream trust-use pathways remain unproven.

Third, the sensor-impairment mini-grid evaluates RS-MARL and Safe-MAPPO under selected packet-loss, observation-noise, and one-step message-delay settings. RS-MARL has descriptively lower mean formation error and higher return than Safe-MAPPO in both the benign and collusive domains, but no comparison survives Holm correction (Table 11). This block supports external-validity checking under modest communication and sensing degradation; it is not a full claim of robustness to arbitrary network impairment.

Finally, the n=100 key-domain rerun retests collusive attacks at 10% and 30% Byzantine ratios and sign-flip at 20%. The high-seed block reduces uncertainty but does not produce Holm-significant RS-MARL versus Safe-MAPPO differences in safety, formation error, or return (Table 12). This result reinforces the conservative statistical interpretation of the core matrix: the absence of corrected significance should not be reported as superiority, even in domains selected for higher statistical power.

### 6.6. Main Result: Safety-Oriented Robustness

**Figure 1:**
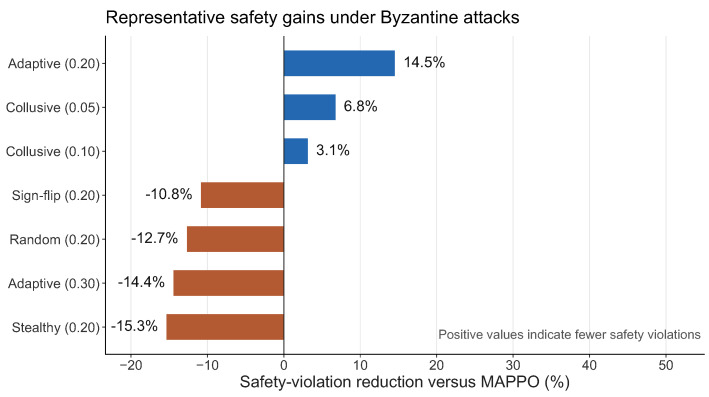
Selected safety-violation differences between RS-MARL and MAPPO. Positive values indicate fewer safety violations for RS-MARL; blue bars show positive reductions and brown bars show negative reductions. The figure is intended to summarise interpretable regimes rather than claim uniform dominance. Corrected statistical interpretation should be taken from the full Holm-audited tables rather than from visual bar height alone.

Figure 1 plots selected safety-violation differences between RS-MARL and MAPPO. The full matrix shows a setting-dependent pattern rather than uniform advantage: RS-MARL reduces mean safety violations relative to MAPPO in 8 of 30 attack-ratio cells, including adaptive, collusive, sign-flip, and stealthy settings, but it is worse in several constant, random, and stealthy cells. After Holm correction, none of the core safety comparisons reaches family-wise significance. We therefore interpret these results as evidence for conditional safety-oriented behaviour, not as evidence of broad dominance over MAPPO or the strongest non-RS baseline. Table 13 reports selected paired safety tests with Holm-adjusted values, and Table 14 lists representative safety-reduction cells together with a counterexample.

**Figure 2:**
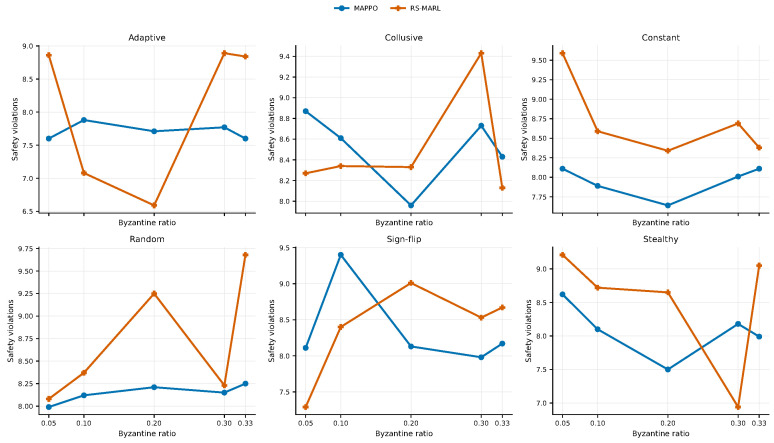
Safety violations versus Byzantine ratio across all attack types for MAPPO and RS-MARL. The comparison is setting-dependent: RS-MARL is safer in some attack-ratio cells but not uniformly better across the full matrix. The figure helps identify candidate operating regimes; it does not by itself establish family-wise statistical superiority.

Figure 2 further shows safety violations across Byzantine ratios and attack types. The curves make clear that attack family and Byzantine ratio jointly determine whether trust-aware safety filtering is beneficial.

### 6.7. Safety-Performance Trade-Off

**Figure 3:**
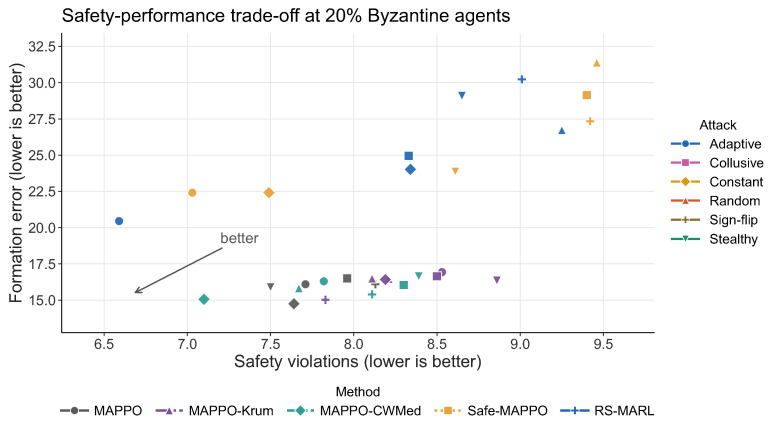
Safety-performance trade-off at a 20% Byzantine ratio. Each point represents one method under one attack setting. Colours distinguish attack families, and marker shapes distinguish methods. Lower-left is better. RS-MARL often exhibits higher formation error, and its safety advantage is setting-dependent. This plot is intended to make the practical cost of defensive conservatism visible alongside safety outcomes.

Figure 3 shows that RS-MARL frequently pays for safety-oriented filtering with higher formation error and lower return. Table 15 reports selected favourable cells together with a random-attack counterexample, making the trade-off visible rather than selecting only positive cases.

### 6.8. Detection Performance

**Figure 4:**
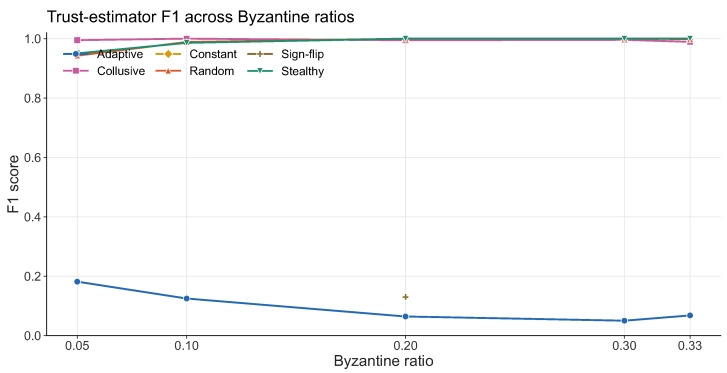
Detection F1 score of the RS-MARL trust estimator across Byzantine ratios. Detection is strong under collusive, random, and stealthy attacks, but weak or undefined under adaptive, sign-flip, and constant attacks. Constant, adaptive, and sign-flip panels mark detector-limited regimes in which RS-MARL should not be treated as a standalone defence.

Figure 4 shows that the trust estimator is more effective under random, collusive, and stealthy attacks than under adaptive, constant, or sign-flip attacks. This is consistent with the assumption that attacks inducing clearer distributional deviations are easier to detect. Table 16 reports selected high-detection regimes with seed-level 95% confidence intervals. These scores should be interpreted as soft reliability evidence rather than definitive Byzantine labels. For practical use, the key limitation is that constant, adaptive, and sign-flip attacks are not detector-supported regimes for the current RMS-energy feature; additional offset-sensitive, sign-sensitive, or direction-sensitive detection is required before the pipeline can be relied on as the sole defence in those settings.

### 6.9. Core Baseline Slice

**Figure 5:**
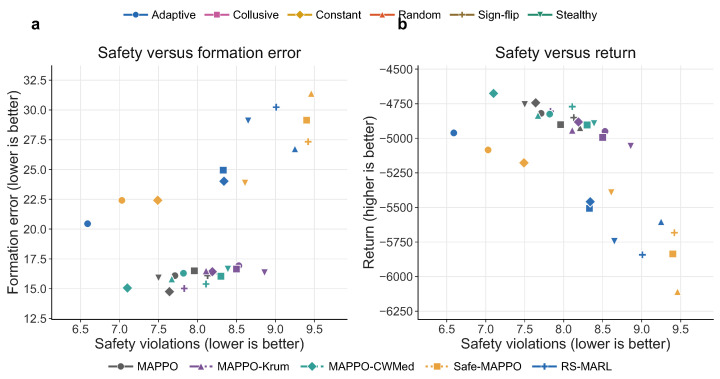
Core-method comparison at a 20% Byzantine ratio using only the audited 3000-run matrix. Each point is one method-attack cell. Colours distinguish attack families, and marker shapes distinguish methods. (**a**): Safety versus formation error. (**b**): Safety versus return. Robust aggregation and safety filtering preserve different properties under Byzantine stress, so no single method uniformly dominates both axes. The slice is descriptive; scope claims are based on the complete matrix and corrected tests.

Figure 5 shows the 20% Byzantine-ratio slice of the audited core matrix without mixing in any legacy ablation rows. The slice is illustrative rather than decisive: robust-aggregation baselines preserve tracking or return in some attack families, whereas safety-oriented baselines more directly target constraint violations. Across the full matrix, however, RS-MARL does not uniformly dominate these baselines. This is why the operational-scope analysis below reports per-family detection, cell-wise safety wins, and Holm-adjusted significance rather than a single aggregate ranking.

### 6.10. Operational Scope and Boundaries of Applicability

To make the practical implications of the above results concrete, we summarise the operational scope of RS-MARL along three axes: (i) detection capability per attack family, (ii) the number of (attack, Byzantine-ratio) cells in which RS-MARL achieves strictly lower mean safety violations than each baseline, and (iii) family-wise significance under Holm correction across all paired comparisons. The summary is built from the completed full 5×6×5×20 matched matrix (3000 runs) provided in the validated data package.

Table 17 consolidates Table 18, Table 19 and Table 20 into a single decision-oriented view. Three evidence regimes emerge. *Most defensible operating regime*: under collusive attacks, Byzantine detection is essentially saturated (F1 ≈0.99), and mean safety violations are lower in 3 of 5 ratio cells against MAPPO. *Detection-rich but safety-conditional regimes*: under random and stealthy attacks, detection remains high (attack-family mean F1 > 0.98), but descriptive safety reductions against the strongest baselines occur only in a minority of cells; use decisions should therefore be governed by application-specific safety budgets. *Detector-limited regimes*: under constant, adaptive, and sign-flip attacks, detection is weak or undefined (constant recall =0; adaptive F1 ≤0.13 across ratios; sign-flip F1 ≤0.13 where defined), and lower mean safety violations against MAPPO occur in at most 2 of 5 ratios. In these regimes, RS-MARL should be paired with an attack-specific detector rather than used as the sole defence. This detector-limited category is treated as a central limitation of the present pipeline, not as a minor exception.

Across all 360 paired comparisons (Table 20), 65 reach raw p<0.05, but none survive Holm correction. The minimum adjusted value is 0.0579 under metric-family correction and 0.1737 under the stricter global audit. We report this transparently rather than emphasising raw-*p* wins because, at this multiplicity, family-wise significance is the correct admissibility criterion. Combined with the safety-cell view of Table 19, RS-MARL offers a consistent local safety advantage over Safe-MAPPO (19/30 cells, negative mean Δ) and a narrower advantage over MAPPO and the robust-aggregation baselines (MAPPO 8/30 cells, MAPPO-Krum 9/30 cells, and MAPPO-CWMed 5/30 cells). This pattern is consistent with the safety-oriented interpretation we adopt throughout the paper.

**Figure 6:**
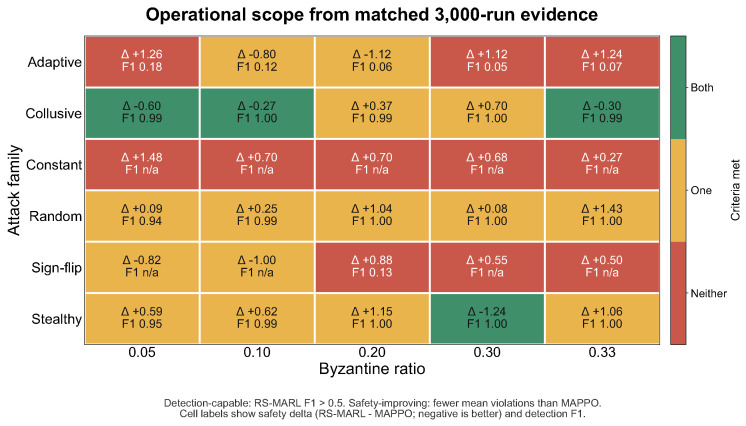
Operational scope of RS-MARL per (attack, Byzantine-ratio) cell. Cells are coloured by joint satisfaction of (i) Byzantine-detection F1 > 0.5 and (ii) lower mean safety violations than MAPPO. Cell labels report the safety-violation delta Δ= RS-MARL − MAPPO (negative is better). The heatmap is intended as a decision aid that complements the aggregate tables. Green, amber, and red cells should be read as supported, partial, and outside-envelope regimes, respectively, not as proof of universal algorithmic dominance.

The applicability heatmap in Figure 6 renders the same information at the (attack, ratio) cell level: green cells indicate that RS-MARL is jointly safety-improving and detection-capable, amber cells indicate that one criterion is satisfied, and red cells indicate that neither criterion is satisfied. The figure makes the regime structure visible: collusive attacks are predominantly green, random and stealthy form an amber band, and constant, sign-flip, and adaptive attacks contain the red regions where RS-MARL lies outside the current operating envelope unless additional safeguards are added.

The boundaries delineated above do not contradict the safety-oriented design of RS-MARL; they are the empirically supported limits within which the design assumptions hold. Future work should target the red regions either by extending the trust estimator to capture constant-bias, adaptive-direction, and sign-flip signatures or by composing RS-MARL with an attack-specific detector dedicated to those families.

## 7. Discussion

### 7.1. Safety-Oriented Robustness Rather than Universal Performance Improvement

The results support a safety-oriented interpretation of RS-MARL rather than a universal performance-improvement claim. Across the full 3000-run matched matrix, RS-MARL has lower mean safety violations in selected Byzantine attack settings compared with MAPPO, with the clearest operating regime under collusive attacks in Table 17. No safety comparison remains significant after Holm correction, so the evidence should be read as conditional and cell-level rather than as family-wise statistical dominance. The fresh benign-control rerun strengthens this boundary: under no attack, RS-MARL is not the strongest nominal controller in formation error, return, or safety violations. This distinction matters: the method is treated here as a bounded safety-oriented design with explicit failure regimes and nominal-regime costs, not as a broadly superior controller across all methods and metrics. This is why the manuscript is framed as an evaluation and auditing study: its main outcome is a disciplined map of supported and unsupported claims, not an assertion of broad algorithmic superiority.

The same results also show that safety gains can come at a cost. RS-MARL often produces larger formation error than MAPPO or robust aggregation baselines, and it does not consistently improve return. This behaviour is consistent with the design objective. Trust down-weighting changes the information available for coordination, while A-CBF action correction can override tracking-favourable actions when constraints become active. The method is therefore most appropriate when reducing unsafe interactions is more important than preserving nominal formation accuracy.

### 7.2. Understanding the Safety-Performance Trade-Off

The safety-performance trade-off has two interacting sources. First, trust-based weighting can suppress corrupted information, but false positives may also reduce useful signals from benign agents. Second, the safety filter reduces violations by changing the executed action, which can move the agent away from a formation-error-minimising trajectory. These mechanisms are beneficial when safety constraints are binding, but they can be conservative when the nominal policy would otherwise coordinate effectively.

The canonical component-ablation rerun first exposed two interpretation risks: the original no-TWCC switch was coupled with broader downstream trust use, and removing the original A-CBF filter descriptively improved formation error and return in the tested cells. The review-audit extension narrows both risks. The A-CBF calibration scan does not identify a Holm-significant alternative to the deployed $g_{m}=0.55$, and the four-switch ablation separates critic-loss weighting, neighbour-feature trust weighting, and safety-margin trust modulation. In that stronger ablation, only SET removal produces a corrected detection-F1 loss in collusive regimes; no downstream trust-use switch produces a corrected safety, formation-error, or return effect. The conservative interpretation is therefore not that A-CBF is invalid, nor that every trust-use path is independently useful. Rather, the present evidence supports SET as a detector for distribution-shifting attacks and leaves downstream control/critic pathways as plausible but statistically unresolved contributors under the tested budget.

### 7.3. Role and Limitations of Trust Estimation

The statistical-energy trust estimator is most informative when attacks create clear distributional shifts, as seen under collusive, random, and stealthy perturbations. It is less reliable under adaptive, constant, and sign-flip attacks, which can resemble benign deviations or produce weak separation from normal formation dynamics. This asymmetry explains why detection performance alone does not translate into uniform safety improvement across all attack families.

These observations constrain how trust should be used. Trust scores are better interpreted as soft reliability weights than as definitive Byzantine labels. A high-recall detector can still harm coordination if it down-weights benign interactions too aggressively. For this reason, detection metrics should not be reported in isolation; they must be interpreted together with safety violations, formation error, and return.

### 7.4. Threats to Validity and Future Directions

Several limitations define the boundary of the present evidence. First, the benchmark is simulated with fixed observation and action dimensions; heterogeneous agents, high-fidelity simulators, and hardware-in-the-loop settings remain to be tested. The sensor-impairment mini-grid covers packet loss, measurement noise, and one-step delay, but only for RS-MARL and Safe-MAPPO in selected benign and collusive domains; it should not be generalised to arbitrary network impairments or heterogeneous sensor modalities. Second, the baseline set is limited to MAPPO-family variants with robust-aggregation and safety-filtering extensions. We did not compare against Byzantine-resilient MARL methods that use fundamentally different architectures (e.g., MADDPG-based or QMIX-based robust aggregation), Bayesian-game-theoretic approaches, or pure model-based CBF controllers without reinforcement learning. Accordingly, the evidence should not be read as a claim that RS-MARL dominates the full space of Byzantine-resilient MARL or model-based safe-control alternatives. These baselines would provide additional reference points for the operating-regime analysis and should be included in future work. Third, the canonical and four-switch ablations cover selected attack families and Byzantine ratios rather than every core attack-ratio cell. Fourth, no core safety/performance comparison remains significant after Holm correction, even when the cell-level direction is favourable; power/sensitivity calculations indicate that the n=20 core cells are underpowered for small effects, and the n=100 key-domain audit still does not produce corrected superiority claims. Fifth, the trust estimator remains structurally weak under constant, adaptive, and sign-flip attacks, as analysed in Section 7.5. These failures are particularly important because they restrict practical deployment unless the detector is extended beyond RMS-energy statistics.

These limitations point to concrete next steps. The most direct technical improvement is to augment the RMS-energy trust feature with offset-sensitive, sign-sensitive, and direction-sensitive statistics, because the completed review-audit confirms that detection, rather than A-CBF margin tuning alone, is the binding constraint in constant, adaptive, and sign-flip regimes. A second priority is to evaluate fundamentally different robust-MARL and model-based safety baselines under the same multiplicity-corrected protocol. A third priority is to expand the sensor-impairment grid to heterogeneous sensing, longer delays, intermittent connectivity, and hardware-in-the-loop execution. Finally, learning-curve and per-seed distribution visualisations should be retained as diagnostic artefacts in future submissions because they make instability visible even when final mean comparisons are inconclusive.

### 7.5. Detector-Limited Regimes Under Constant, Adaptive, and Sign-Flip Attacks

The near-zero recall under constant, adaptive, and sign-flip attacks (Table 18) appears to reflect a structural limitation of the RMS-energy trust feature rather than only a parameter-tuning issue. Under random, collusive, or stealthy attacks, Byzantine messages exhibit distributional shifts from the benign message manifold, such as inflated variance, coordinated displacement, or constrained subspace deviation. The sliding-window RMS-energy statistic can amplify these shifts when the within-window energy of Byzantine messages differs from benign messages. Under a constant attack, however, a fixed additive bias shifts the sliding-window mean but does not necessarily increase within-window RMS energy; once the window has absorbed the offset, the anomaly score remains close to the benign baseline, which is consistent with recall =0. Under an adaptive attack, Byzantine perturbations are aligned with each agent’s target-relative direction, which varies smoothly as the formation converges. The per-agent message variance under adaptive attack is difficult to separate from that of a benign agent undergoing formation correction. Under sign-flip attacks, the magnitude of each message component is preserved; only the sign changes. The RMS-energy statistic, which depends on squared deviations from the window mean, is insensitive to this sign change. In these cases, the energy feature remains close to the benign baseline, the median/MAD-normalised anomaly score $z_{ij}^{t}$ approaches zero, and the trust score $\tau_{ij}^{t}$ remains near one regardless of Byzantine status.

This detector-limited regime is expected for trust estimators that rely solely on message-energy statistics. Addressing it requires either (i) augmenting the feature set with offset-sensitive, sign-sensitive, or direction-sensitive statistics (e.g., mean-shift persistence, message-velocity consistency, cross-agent prediction residuals), or (ii) coupling the RMS-energy estimator with an attack-specific detector for the families where energy features are non-informative. We leave this augmentation to future work and note that the very low or undefined F1 under constant, adaptive, and sign-flip attacks is a major empirical constraint on RS-MARL’s applicable regime. In application terms, these attacks should be treated as outside-envelope cases for the current implementation, not as settings where the reported safety reductions can be generalised.

### 7.6. Scope and Interpretation of Claims

To prevent over-interpretation, we state the scope of the reported evidence explicitly:

1. **Scope of safety improvement.** RS-MARL reduces mean safety violations in selected attack-ratio cells, but the advantage is setting-dependent and is not reported as family-wise statistical dominance after multiplicity correction.
2. **Scope of detection.** The SET trust estimator is informative under distribution-shifting attacks (collusive, random, stealthy) but provides weak or undefined detection under adaptive, sign-flip, and constant attacks. It should therefore be used as a soft reliability signal rather than as a standalone Byzantine detector.
3. **Scope of nominal operation.** Under no Byzantine attack, RS-MARL does not set the best mean formation error, return, or safety-violation metric. These benign-control differences are interpreted as a descriptive nominal-cost audit, not as Holm-significant degradation claims.
4. **Scope of module attribution.** The four-switch ablation separates SET, critic-loss weighting, neighbour-feature trust weighting, and safety-margin trust modulation. It supports SET as independently necessary for collusive-attack detection, but it does not establish Holm-significant safety, formation-error, or return contributions for every downstream trust-use path.
5. **Scope of algorithmic novelty.** SET, TWCC, and A-CBF each adapt known techniques (sliding-window anomaly detection, trust-weighted aggregation, CBF-inspired filtering). The contribution lies in their integration as a representative safety pipeline, the audited 3000-run core matrix, the 2960 supplemental and review-audit runs, and the operating-regime evaluation framework, not in claiming a new optimisation or control primitive. This is the intended positioning of the manuscript as an evaluation and audit study.

These scope statements define the conditions under which the reported evidence can be interpreted and prevent the results from being cited as evidence for claims the data do not support.

### 7.7. Reproducibility and Artefact Audit

The numerical tables in this version are computed from per-seed CSV summaries rather than manually transcribed values. The scripts integrate_v3_cloud_results.py, q1_evidence_tables.py, and collect_results_v3.py verify the core archive, regenerate group summaries, audit matrix completeness, test missing paired seeds, compute Holm-adjusted significance statistics, and produce the LaTeX table fragments used in this manuscript. The core source archive is rsmarlv3returnkey20260527124029.tar.gz; its SHA256 digest begins 9b5a7f637f29, with the full digest recorded in the checksum file. The core audit reports 3000/3000 successful runs, 150/150 complete cells, and 20 seeds in every method-attack-ratio cell.

The V3 publication data package adds canonical benign and ablation reruns with 100/100 and 480/480 successful runs, respectively, and provides processed summaries, significance-test tables, final figure/table source files, a package README, code snapshot, environment record, logs, and SHA256 checksums for 3,196 files. The review-audit V2 return package adds 2380/2380 successful runs with a verified return archive and processed summaries for A-CBF calibration, four-switch ablation, sensor-impairment, and n=100 key-domain audits. Its collection manifest reports zero missing or invalid runs, and the SHA256 digest of the returned archive is recorded in the accompanying checksum file. The code snapshot provides the base training configuration, attack parameters, and trust/safety constants summarised in Table 2, Table 3 and Table 4. Per-run configuration files record the method, attack, ratio, seed, episode count, horizon, and review-audit modifiers. The supporting data package is cited as File S1 in the Supporting Information.

## 8. Conclusions

This paper presents a multiplicity-corrected operating-regime evaluation of Byzantine-resilient MARL for sensor-networked safe formation control, instantiated on a representative trust-based safety pipeline. The work should be read as an evaluation and auditing contribution rather than as a claim that RS-MARL is a broadly dominant new algorithm. The audited evidence base combines a 3000-run core matrix, 580 canonical supplemental runs, and a 2380-run review-audit extension. It uses matched seeds, Holm correction over 360 core contrasts, per-cell completeness auditing, SHA256-verified artefacts, benign-control, component-ablation, calibration, sensor-impairment, and high-seed reruns. The central lesson is that robustness claims require more than favourable cell-wise comparisons: they require multiplicity-corrected significance, nominal-regime cost measurement, detection-reliability characterisation by attack family, and explicit acknowledgement of the safety-performance trade-off.

The RS-MARL case study demonstrates why this discipline matters. Under collusive, random, and stealthy attacks where distribution-shifting signatures are detectable, the pipeline can reduce safety violations in selected baseline comparisons. Under constant, adaptive, and sign-flip attacks, the present energy-based trust feature is not sufficiently discriminative for reliable trust weighting. These three attack families therefore remain practical failure regimes for the current detector and require additional offset-sensitive, sign-sensitive, or direction-sensitive detection before standalone deployment. Under no attack, RS-MARL does not set the best nominal metrics, which quantifies the cost of conservative defence. The review-audit extension further shows that simple A-CBF margin retuning does not yield a corrected improvement over the deployed setting, and that SET is the only component with a Holm-significant independent effect, specifically on collusive-attack detection. These findings are not presented as universal method superiority; they define the operating regimes in which the design assumptions are supported and the regimes in which additional sensing or detection mechanisms are needed.

We recommend that future Byzantine-resilient MARL studies adopt a more explicit reporting template: (i) a complete seed-matched matrix spanning methods, attacks, and ratios; (ii) multi-comparison-corrected significance testing with explicit reporting of both raw and adjusted *p*-values; (iii) a benign-control block quantifying nominal-regime defence cost; (iv) a same-pipeline component-ablation block that tests both module effects and module coupling; (v) an operating-regime summary identifying where the method is useful, where support remains inconclusive, and where detection is unreliable; and (vi) a clear scope-of-claims statement. This template requires a larger experimental budget than a single-sweep comparison, but it turns a method demonstration into an auditable scientific claim. It also makes negative and inconclusive results visible, which is essential when corrected statistical evidence does not support broad superiority claims.

## Figures and Tables

**Table 1:** Experimental setup for the validated dataset.

Item	Setting
Task	Multi-agent formation control
Agents and dimensions	N=30, 10-dimensional observation, 2-dimensional continuous action
Training schedule	50 training iterations with 256 rollout steps
Evaluation episodes	5 final evaluation episodes per seed
Episode horizon	250 environment steps
Random seeds	20
Attack types	Constant, random, sign-flip, stealthy, adaptive, collusive
Byzantine ratios	Nominal ratios 0.05, 0.10, 0.20, 0.30, 0.33; with N=30, nearest-integer compromised-agent counts are 2, 3, 6, 9, and 10, giving realised ratios 0.067, 0.100, 0.200, 0.300, and 0.333
Core methods	MAPPO, Safe-MAPPO, MAPPO-Krum, MAPPO-CWMed, RS-MARL
Supplemental studies	Benign-control rerun (100/100), canonical component-ablation rerun (480/480), and review-audit V2 extension (2380/2380)
Current validated runs	5960/5960 across core and supplemental evidence blocks
Summary groups	150 core cells, 5 benign-control cells, 24 canonical ablation cells, and four review-audit V2 blocks
Final metric window	Mean over the 5 final evaluation episodes for each seed

**Table 2:** Implementation and hyperparameters used by the validated training configuration and runner manifest.

Category	Item	Value/Source
Environment	Formation task	30 agents, dt=0.05, world size 16.0, lattice spacing 3.0, communication radius 6.0
Safety geometry	Thresholds and bounds	Safe distance 0.75, obstacle radius 1.2 at (0,0), maximum acceleration 2.0
Training schedule	Episodes, horizon, seeds	50 training iterations, 256 rollout steps, 5 evaluation episodes, seeds 0–19
Policy/value networks	Architecture and activation	Shared-parameter actor and critic; two hidden layers, 128 units each, Tanh activations; learned diagonal Gaussian log standard deviation
Optimisation	Optimiser and batch	Adam, learning rate $10^{-4}$, minibatch size 1024, four PPO epochs per update
MAPPO/PPO settings	Discount, GAE lambda, clip ratio	γ=0.99, GAE λ=0.95, clip ϵ=0.10
Loss weights	Entropy, value, gradient clipping	Entropy coefficient 0.005, value coefficient 0.50, gradient norm clipped at 0.5
Software	Core dependencies	Python with NumPy, SciPy, PyTorch, pandas, Matplotlib, seaborn, and PyYAML; software versions are documented in the supplementary environment record (File S1); CPU execution is supported

**Table 3:** Attack parameters used by the released attack implementation.

Attack	Perturbed Signal	Perturbation Rule	Parameters
Constant	Message position components	Add fixed positive bias to the first two message dimensions	Magnitude 2.0
Random	Message position and velocity components	Replace position components by uniform noise and velocity components by Gaussian noise	Uniform [−2,2], Gaussian $N(0,2^{2})$
Sign-flip	Selected message components	Multiply configured components by −1	Components 0–3
Stealthy	Message vector	Sample perturbations in the leading subspace of honest-message deviations	Stealth strength 0.8; leading two singular directions
Adaptive	Message position components	Move Byzantine messages away from their target direction	Magnitude 2.0 with normalised target-relative direction
Collusive	Byzantine message positions	Replace Byzantine positions by a shared phantom target with small noise	Phantom offset (2,−2), noise $N(0,0.05^{2})$

**Table 4:** Safety and trust parameters used by the released implementation.

Module	Item	Value/Source
Safety constraints	Collision, obstacle, connectivity definitions	Collision if pairwise distance is below 0.75; obstacle violation if distance to the obstacle boundary is below 0.75; connectivity violation if nearest-neighbour distance exceeds 6.0
A-CBF	Execution-time correction	Vectorised CBF-inspired repulsive correction with trust-dependent margins $0.75+0.55(1-\tau_{ij})$, obstacle repulsion, and action clipping at norm 2.0
A-CBF	Logged slack proxy	Sum of positive pairwise margin deficits; this quantity is a violation proxy rather than the slack variable of a solved QP
SET	Trust scoring	Sliding window of 12 message histories, RMS-energy features, median/MAD normalisation, and exponential reliability exp(−z/T) with T=3.0
SET	Trust update and detection	Exponential smoothing 0.15; Byzantine prediction threshold 0.5 on receiver-averaged trust
TWCC	Trust normalisation and weighting rule	Receiver-side trust weights are normalised over neighbours for message aggregation; sender trust is averaged across receivers and clipped to [0.05,1.0] as the critic-loss weight during MAPPO updates
Validation	Run-status and missing-pair audit	Produced from q1_evidence_tables.py and audit_experiment_archive.py

**Table 5:** Current completeness of the core matched-comparison matrix. A full core matrix requires 30 attack-ratio groups and 600 per-seed runs for each method.

Method	Complete Groups	Runs	Expected	Missing Groups
MAPPO	30/30	600	600	0
MAPPO-Krum	30/30	600	600	0
MAPPO-CWMed	30/30	600	600	0
Safe-MAPPO	30/30	600	600	0
RS-MARL	30/30	600	600	0

**Table 6:** Evidence inventory after integrating the V3 publication data package and the review-audit V2 extension. The core adversarial matrix is sourced from the return_key archive timestamped 20260527_124029 (SHA256 prefix 9b5a7f637f29) and supports baseline comparisons. The canonical benign and ablation matrices provide nominal-regime and first-pass component audits. The review-audit V2 block adds A-CBF calibration, four-switch ablation, sensor-impairment, and high-seed confirmation experiments.

Evidence Block	Successful Runs	Complete Cells	Role in Claims
Core adversarial matrix	3000/3000	150/150	Main evidence for baseline comparisons, operating regimes, detection reliability, and multiplicity-corrected statistics.
Benign-control rerun	100/100	5/5	Tests nominal-regime cost of robustness and safety filtering under no Byzantine attack.
Canonical component-ablation rerun	480/480	24/24	First-pass module audit for adaptive, collusive, and constant attacks at ratios 0.10 and 0.30.
Review-audit V2 extension	2380/2380	2380 per-run jobs	A-CBF calibration, four-switch component audit, sensor-impairment mini-grid, and n=100 key-domain confirmation.

**Table 7:** Fresh benign-control rerun under the V3 pipeline. Values are mean ± 95% confidence-interval half-width over 20 seeds. Lower formation error and safety violations are better, while higher return is better. Downward and upward arrows indicate lower-is-better and higher-is-better columns, respectively.

Method	Formation Error ↓	Return ↑	Safety Violations ↓
MAPPO	**15.513** ± 2.127	−4867.494 ± 295.611	7.910 ± 1.753
MAPPO-CWMed	15.516 ± 2.069	**−4804.122** ± 289.737	**7.640** ± 1.588
MAPPO-Krum	16.471 ± 2.409	−4880.681 ± 260.982	8.640 ± 1.525
RS-MARL	31.424 ± 12.342	−5939.726 ± 1131.984	8.770 ± 2.279
Safe-MAPPO	32.763 ± 12.917	−6238.321 ± 1009.250	9.600 ± 2.149

**Table 8:** Component-ablation directionality in the fresh V3 supplemental rerun. Counts show the number of attack-ratio cells in which full RS-MARL is better than the ablated variant. Downward and upward arrows indicate lower-is-better and higher-is-better metrics, respectively. The ablation set contains adaptive, collusive, and constant attacks at Byzantine ratios 0.10 and 0.30, with 20 seeds per cell. The no-SET and no-TWCC variants are numerically indistinguishable in this data block; because the current no-TWCC switch suppresses downstream trust use broadly, the table supports a coupling audit rather than independent attribution of a critic-only pathway. No ablation comparison survives Holm correction.

Comparison	FE Better ↓	Return Better ↑	Safety Better ↓	Detection F1 Better ↑
RS-MARL vs. RS-MARL-no-A-CBF	0/6	0/6	1/6	1/4
RS-MARL vs. RS-MARL-no-SET	5/6	5/6	4/6	n/a
RS-MARL vs. RS-MARL-no-TWCC	5/6	5/6	4/6	n/a

**Table 9:** A-CBF calibration scan over adaptive margin gain $g_{m}$. Values average the seven E1 cells (benign plus adaptive, collusive, and sign-flip attacks at 10% and 30% Byzantine ratios), with 20 seeds per cell. Lower is better for safety violations and formation error; higher is better for return and detection F1. The sign-flip F1 average is defined only over cells where the detector produced valid positives. No comparison against the deployed $g_{m}=0.55$ survives Holm correction (minimum raw p=0.0144; all adjusted p=1.000).

$g_{m}$	Safety Violations	Formation Error	Return	Detection F1
0.00	8.78	26.95	−5644.9	0.286
0.10	8.79	26.83	−5620.4	0.289
0.20	8.76	27.46	−5669.7	0.292
0.35	8.89	26.88	−5641.0	0.287
0.55	8.49	25.23	−5477.8	0.288

**Table 10:** Four-switch component audit. Values average the six E2 attack-ratio cells (adaptive, collusive, and sign-flip at 10% and 30% Byzantine ratios), with 20 seeds per cell. Only the SET-disabled detection-F1 losses in collusive regimes remain significant after Holm correction; no safety, formation, or return comparison is significant.

Variant	Safety Violations	Formation Error	Return	Detection F1
Full	8.44	24.20	−5400.9	0.336
No SET	8.16	25.15	−5456.2	0.000
No TWCC-critic	8.58	24.63	−5488.6	0.337
No TWCC-feature	8.32	24.84	−5409.0	0.331
No A-CBF trust margin	8.46	27.14	−5646.1	0.339

**Table 11:** Sensor-impairment mini-grid summary. Values average 12 impairment settings (packet loss, observation noise, and one-step message delay), with 10 seeds per setting. These results support external-validity auditing but are not significant after Holm correction (minimum raw p=0.0137; all adjusted p≥0.984).

Domain	Method	Safety Violations	Formation Error	Return
collusive, 0.3	RS-MARL	8.89	25.02	−5483.5
collusive, 0.3	Safe-MAPPO	9.09	27.39	−5722.6
benign	RS-MARL	9.12	25.80	−5514.4
benign	Safe-MAPPO	9.18	27.45	−5694.4

**Table 12:** Key-domain n=100 audit comparing RS-MARL and Safe-MAPPO. The high-seed rerun reduces uncertainty but does not produce Holm-significant safety, formation, or return differences in the three audited domains.

Attack	Ratio	RS Safety	Safe Safety	RS FE	Safe FE	RS Return	Safe Return	Safety adj. *p*
collusive	0.1	8.15	8.14	22.18	23.13	−5245.1	−5302.2	1.000
collusive	0.3	8.52	8.26	21.77	23.74	−5220.5	−5346.2	1.000
sign-flip	0.2	8.38	7.94	23.48	22.73	−5353.4	−5292.7	1.000

**Table 13:** Seed-level significance tests for selected safety-violation comparisons. Tests use paired Wilcoxon signed-rank tests when matching seeds are available; $p_{\mathrm{adj}}$ is Holm-adjusted across safety comparisons. Negative Δ indicates fewer safety violations for RS-MARL. n.s. denotes not significant after Holm correction.

Baseline Method	Attack	Ratio	Baseline Viol.	RS-MARL Viol.	Δ	*d*	$p_{\mathrm{adj}}$
MAPPO	adaptive	0.20	7.710	6.590	−1.120	−0.206	1.000 n.s.
MAPPO	adaptive	0.30	7.770	8.890	1.120	0.204	1.000 n.s.
MAPPO	collusive	0.05	8.870	8.270	−0.600	−0.125	1.000 n.s.
MAPPO	collusive	0.10	8.610	8.340	−0.270	−0.042	1.000 n.s.
MAPPO	random	0.20	8.210	9.250	1.040	0.237	1.000 n.s.
MAPPO	sign-flip	0.20	8.130	9.010	0.880	0.136	1.000 n.s.
MAPPO	stealthy	0.20	7.500	8.650	1.150	0.242	1.000 n.s.
MAPPO-Krum	collusive	0.05	8.290	8.270	−0.020	−0.004	1.000 n.s.
MAPPO-Krum	collusive	0.10	8.120	8.340	0.220	0.043	1.000 n.s.
Safe-MAPPO	adaptive	0.20	7.030	6.590	−0.440	−0.131	1.000 n.s.
Safe-MAPPO	collusive	0.20	9.400	8.330	−1.070	−0.235	1.000 n.s.

**Table 14:** Selected safety-violation comparisons between MAPPO and RS-MARL. Values are mean ± 95% confidence intervals over 20 paired seeds. Positive reduction indicates fewer safety violations for RS-MARL; the final row is retained as a counterexample.

Attack	Ratio	MAPPO	RS-MARL	Reduction
adaptive	0.10	7.880 ± 1.366	7.080 ± 1.613	10.2%
adaptive	0.20	7.710 ± 1.679	6.590 ± 1.633	14.5%
collusive	0.05	8.870 ± 1.591	8.270 ± 1.789	6.8%
collusive	0.33	8.430 ± 1.661	8.130 ± 1.862	3.6%
sign-flip	0.05	8.110 ± 1.681	7.290 ± 1.780	10.1%
sign-flip	0.10	9.400 ± 2.187	8.400 ± 2.215	10.6%
stealthy	0.30	8.180 ± 1.793	6.940 ± 1.388	15.2%
random	0.20	8.210 ± 1.595	9.250 ± 2.174	−12.7%

**Table 15:** Safety-performance trade-off between MAPPO and RS-MARL in selected attack-ratio cells. Lower formation error and safety violations are better, while higher return is better.

Attack	Ratio	Method	Formation Error	Return	Safety Violations	Safety Reduction
adaptive	0.20	MAPPO	16.097	−4819.613	7.710	–
adaptive	0.20	RS-MARL	20.454	−4961.199	6.590	14.5%
collusive	0.05	MAPPO	16.098	−4929.317	8.870	–
collusive	0.05	RS-MARL	21.762	−5056.312	8.270	6.8%
sign-flip	0.10	MAPPO	16.620	−5099.144	9.400	–
sign-flip	0.10	RS-MARL	25.044	−5363.152	8.400	10.6%
stealthy	0.30	MAPPO	16.256	−4855.553	8.180	–
stealthy	0.30	RS-MARL	19.939	−4995.643	6.940	15.2%
random	0.20	MAPPO	16.478	−4923.188	8.210	–
random	0.20	RS-MARL	26.735	−5603.058	9.250	−12.7%

**Table 16:** Selected high-detection regimes for the RS-MARL trust estimator with 95% confidence intervals. These rows include all collusive ratios and the high-ratio random and stealthy regimes discussed in the main text.

Attack	Ratio	Precision	Recall	F1
collusive	0.05	0.995 ± 0.009	0.995 ± 0.010	0.995 ± 0.007
collusive	0.10	1.000 ± 0.000	1.000 ± 0.000	1.000 ± 0.000
collusive	0.20	0.992 ± 0.016	0.998 ± 0.003	0.995 ± 0.009
collusive	0.30	1.000 ± 0.000	0.992 ± 0.006	0.996 ± 0.003
collusive	0.33	0.999 ± 0.002	0.980 ± 0.012	0.989 ± 0.006
random	0.20	1.000 ± 0.000	1.000 ± 0.000	1.000 ± 0.000
random	0.30	1.000 ± 0.000	1.000 ± 0.000	1.000 ± 0.000
random	0.33	0.997 ± 0.004	1.000 ± 0.000	0.999 ± 0.002
stealthy	0.20	1.000 ± 0.000	1.000 ± 0.000	1.000 ± 0.000
stealthy	0.30	1.000 ± 0.000	1.000 ± 0.000	1.000 ± 0.000
stealthy	0.33	1.000 ± 0.000	1.000 ± 0.000	1.000 ± 0.000

**Table 17:** RS-MARL operational scope. Detection F1 is averaged over the five Byzantine ratios where defined; the sign-flip F1 value is defined only at the 20% Byzantine ratio because the remaining sign-flip cells are n/a. Safety wins count ratio cells where RS-MARL has lower mean safety violations than MAPPO. Scope labels summarise evidence strength rather than serving as implementation guarantees.

Attack	Detection F1	Wins vs. MAPPO	Evidence Scope
constant	n/a	0/5	*Avoid alone*
random	0.986	0/5	Caution
sign-flip	0.130	2/5	*Avoid alone*
stealthy	0.987	1/5	Caution
adaptive	0.098	2/5	*Avoid alone*
collusive	0.995	3/5	**Best-supported**

**Table 18:** RS-MARL Byzantine detection performance per attack family. Values are averaged over five Byzantine ratios and 20 seeds (n=100 runs per row) where the metric is defined. High F1 on collusive, random, and stealthy attacks is reported alongside weak or undefined detection for constant, adaptive, and sign-flip attacks. For constant attacks, precision and F1 are undefined because the detector produces no positive predictions. The sign-flip F1 value is defined only for the 20% ratio; the remaining sign-flip ratios are n/a, so the value should not be interpreted as a five-ratio mean.

Attack	Precision	Recall	F1
constant	n/a	0.000	n/a
**random**	0.974	1.000	0.986
sign-flip	0.310	0.002	0.130
**stealthy**	0.976	1.000	0.987
adaptive	0.952	0.008	0.098
**collusive**	0.997	0.993	0.995

**Table 19:** Cell-wise safety comparison over the 30 attack-ratio cells. Negative mean Δ indicates fewer safety violations for RS-MARL.

Baseline	Safer Cells	Mean Δ
MAPPO	8/30	+0.354
MAPPO-CWMed	5/30	+0.540
MAPPO-Krum	9/30	+0.180
**Safe-MAPPO**	19/30	−0.230

**Table 20:** Multiplicity audit for all paired comparisons. Metric-family Holm correction is applied within each outcome family (formation error, return, and safety violations); the global Holm row is a stricter audit over all 360 contrasts.

Quantity	Value
Total paired comparisons	360
Comparisons with raw p<0.05	65 (18.1%)
Metric-family Holm p<0.05	**0** (0.0%)
Global Holm p<0.05	**0** (0.0%)
Minimum raw *p*	0.0005
Minimum metric-family Holm *p*	0.0579
Minimum global Holm *p*	0.1737

**Figure A1:**
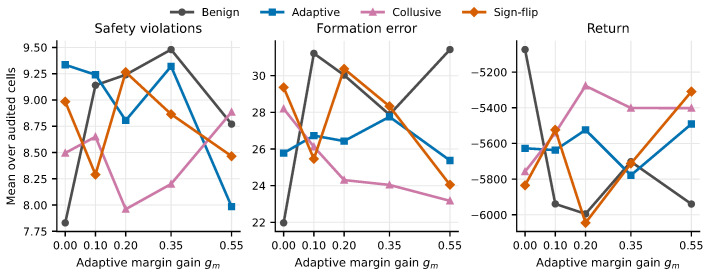
Review-audit V2 A-CBF adaptive-margin calibration. The figure visualises mean safety violations, formation error, and return across tested margin-gain settings. Corrected inference is reported in Table 9; no tested gain significantly dominates the deployed setting after Holm correction.

**Figure A2:**
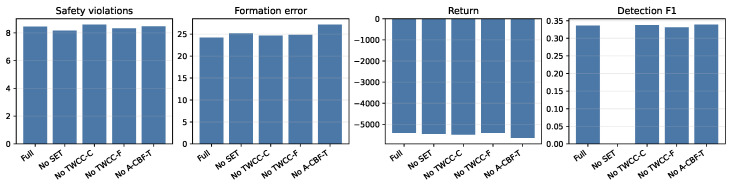
Review-audit V2 four-switch ablation. The plot separates SET, critic-loss weighting, neighbour-feature trust weighting, and trust-dependent safety-margin modulation. Corrected inference is reported in Table 10; the only Holm-significant independent effect is the SET contribution to collusive-attack detection.

**Figure A3:**
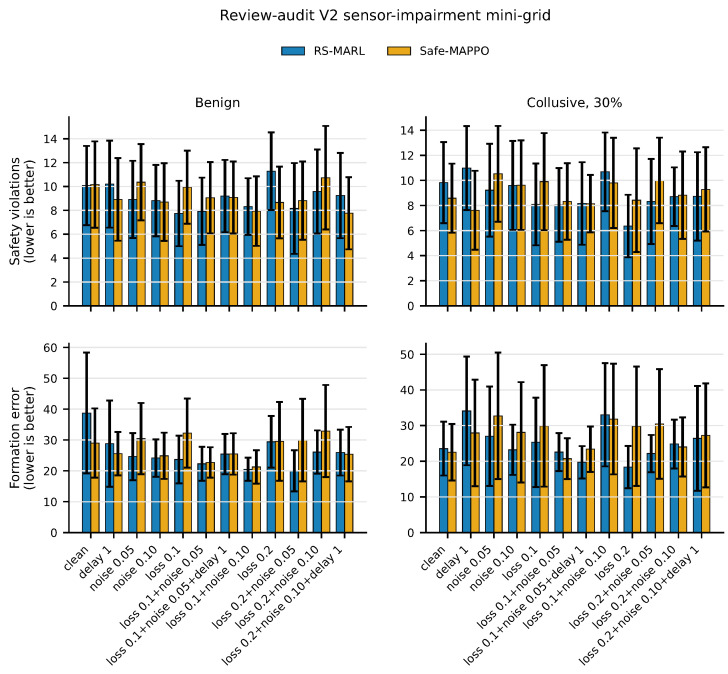
Review-audit V2 sensor-impairment mini-grid. Bars show descriptive means and 95% confidence intervals under selected packet-loss, observation-noise, and one-step delay settings in benign and collusive domains. Corrected inference is reported in Table 11.

**Figure A4:**
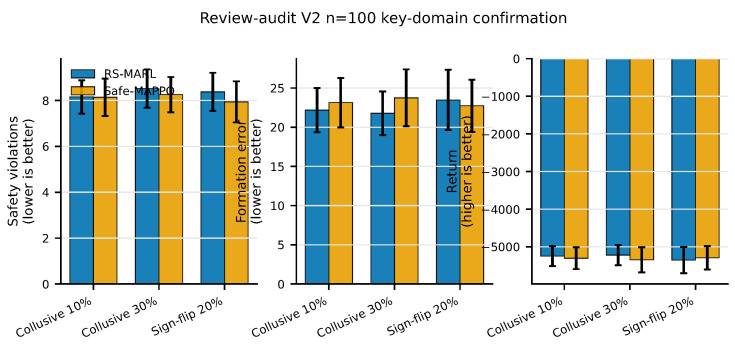
Review-audit V2 n=100 key-domain confirmation. Bars show descriptive means and 95% confidence intervals for collusive attacks at 10% and 30% Byzantine ratios and sign-flip attacks at 20%. Corrected inference is reported in Table 12.

**Figure A5:**
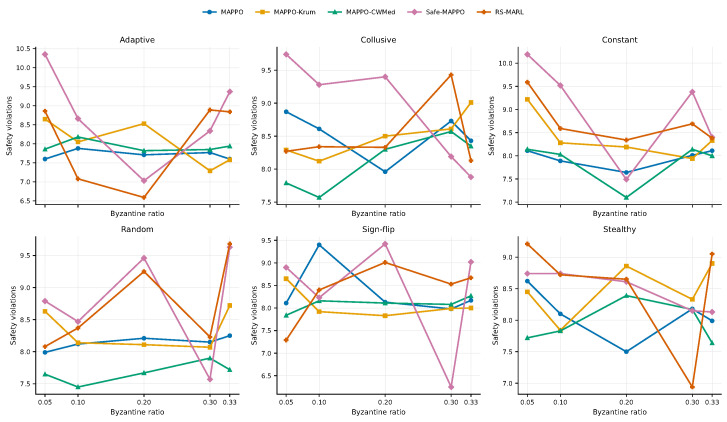
Core-method safety-violation trends across attack types and Byzantine ratios. Lower values are better. The figure is a descriptive source-data view; corrected inference is reported in the main statistical tables.

**Figure A6:**
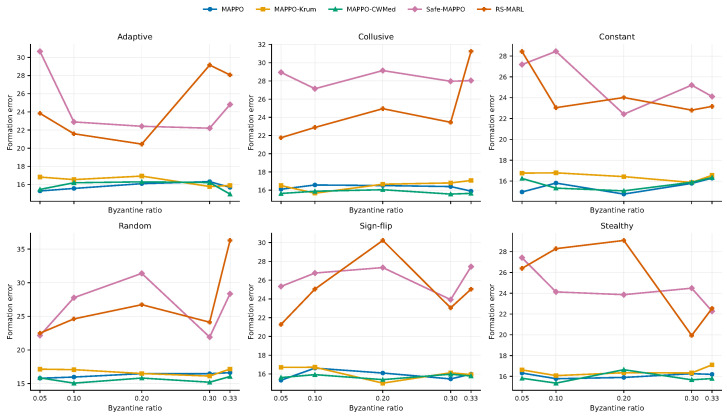
Core-method formation-error trends across attack types and Byzantine ratios. Lower values are better. The figure shows where safety-oriented behaviour coincides with formation-error cost.

**Figure A7:**
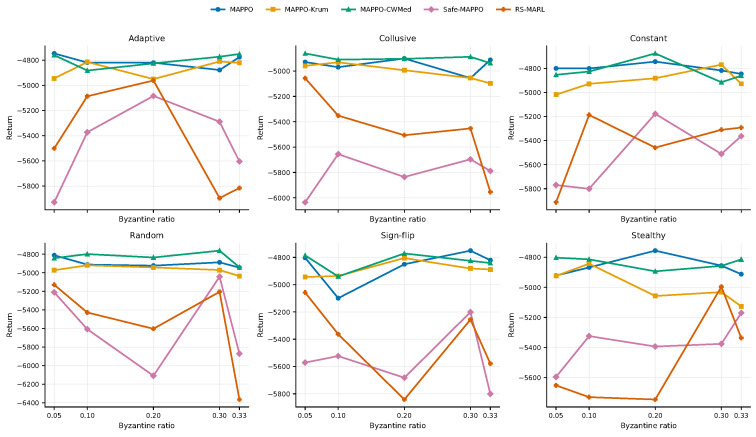
Core-method return trends across attack types and Byzantine ratios. Higher values are better. The figure complements the safety plots by showing that safety gains do not imply uniform return improvement.

**Figure A8:**
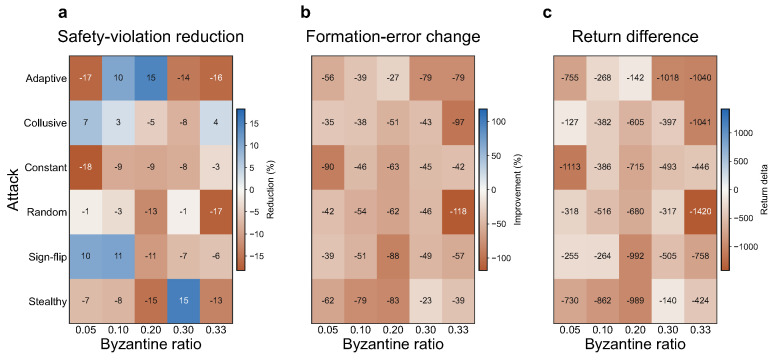
Attack-ratio heatmaps comparing RS-MARL with MAPPO. Positive safety values indicate fewer safety violations for RS-MARL, while formation-error and return panels show the associated performance trade-offs. The heatmaps make operating-regime boundaries visible rather than supporting a single aggregate ranking.

**Figure A9:**
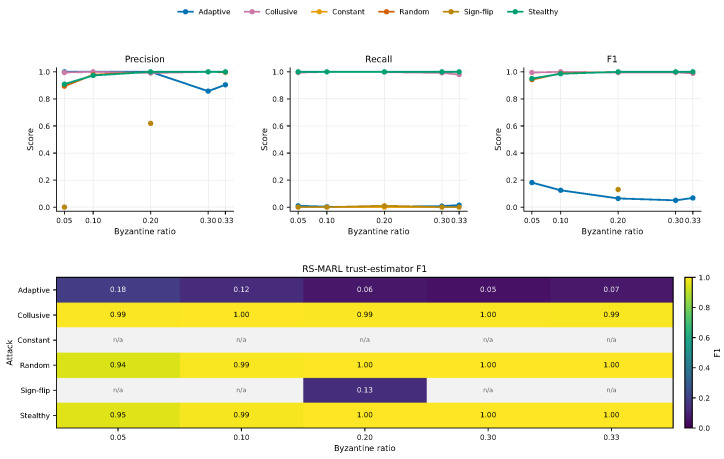
Trust-estimator detection behaviour. The upper panels report precision, recall, and F1 across Byzantine ratios, and the lower heatmap summarises F1 across attack-ratio settings. Low or undefined constant, adaptive, and sign-flip F1 values identify the detector-limited cases that bound practical applicability.

## Data Availability

The processed per-run summaries, statistical-test outputs, analysis tables, figure source data, data dictionaries, and checksum manifests supporting the reported results are included in the supplementary data package prepared with this submission. The complete raw run archives for the 3000-run core matrix, 580-run canonical supplemental block, and 2380-run review-audit V2 extension are provided as separate repository-deposit archives with SHA256 verification and can be supplied to reviewers or deposited in a public repository if required by the journal. No human-participant, clinical, proprietary, or third-party restricted datasets were used.

## Supplementary Materials

The following supporting information can be downloaded at: https://www.mdpi.com/article/10.3390/s26144408/s1. File S1: The following supporting information is provided with the Sensors submission package: processed per-run summaries, completeness audits, Holm-corrected significance tables, LaTeX table fragments, publication figures, data dictionaries, checksum manifests, and repository-deposition instructions for the full raw run archive.

## Abbreviations

The following abbreviations are used in this manuscript:

A-CBF	Adaptive CBF-inspired safety filter
CBF	Control barrier function
CWMed	Coordinate-wise median
GAE	Generalised advantage estimation
Krum	Byzantine-robust aggregation rule
MAD	Median absolute deviation
MADDPG	Multi-agent deep deterministic policy gradient
MARL	Multi-agent reinforcement learning
MAPPO	Multi-agent proximal policy optimisation
PPO	Proximal policy optimisation
QMIX	Monotonic value-factorisation method
QP	Quadratic programme
RMS	Root mean square
RS-MARL	Resilient and safety-oriented multi-agent reinforcement learning
SET	Statistical-energy trust estimator
TWCC	Trust-weighted consensus critic

## Appendix A. Additional Experimental Results

This appendix provides additional plots produced from the full experimental summary. These results complement the main paper by showing trends across attack types, Byzantine ratios, and evaluation metrics.

*Appendix reading guide.* Figure A1, Figure A2, Figure A3 and Figure A4 visualise the 2380-run review-audit V2 extension and should be read together with Table 9, Table 10, Table 11 and Table 12, which provide the corrected statistical interpretation. Figure A5, Figure A6, Figure A7, Figure A8 and Figure A9 should be read as source-data visualisations for the audited core matrix rather than as separate claims. The three trend figures expose how safety violations, formation error, and return vary with attack family and Byzantine ratio. The heatmap figure summarises the same matrix as signed deltas against MAPPO, making clear where safety gains coincide with formation or return costs. The detection figure then shows why these trade-offs are regime-dependent: trust scores are reliable under collusive, random, and stealthy perturbations, but weak or undefined under constant, adaptive, and sign-flip attacks. Together, the appendix supports the main conclusion that RS-MARL should be interpreted through operating regimes, uncertainty, and detection reliability rather than through a single aggregate ranking. Readers should therefore use the appendix to audit the scope of claims, not to infer an uncorrected aggregate winner.
