# Supplementary material for: Operating-Regime Evaluation of Byzantine-Resilient Multi-Agent Reinforcement Learning for Sensor-Networked Safe Formation Control
Source: Sensors (Basel). 2026 Jul 11;26(14):4408. doi: 10.3390/s26144408 (PMC13418820; doi:10.3390/s26144408)
Supplement: Supplementary file 1 [file sensors-26-04408-s001.zip › File_S1/figures/core/fig05_core_baseline_journal.pdf]

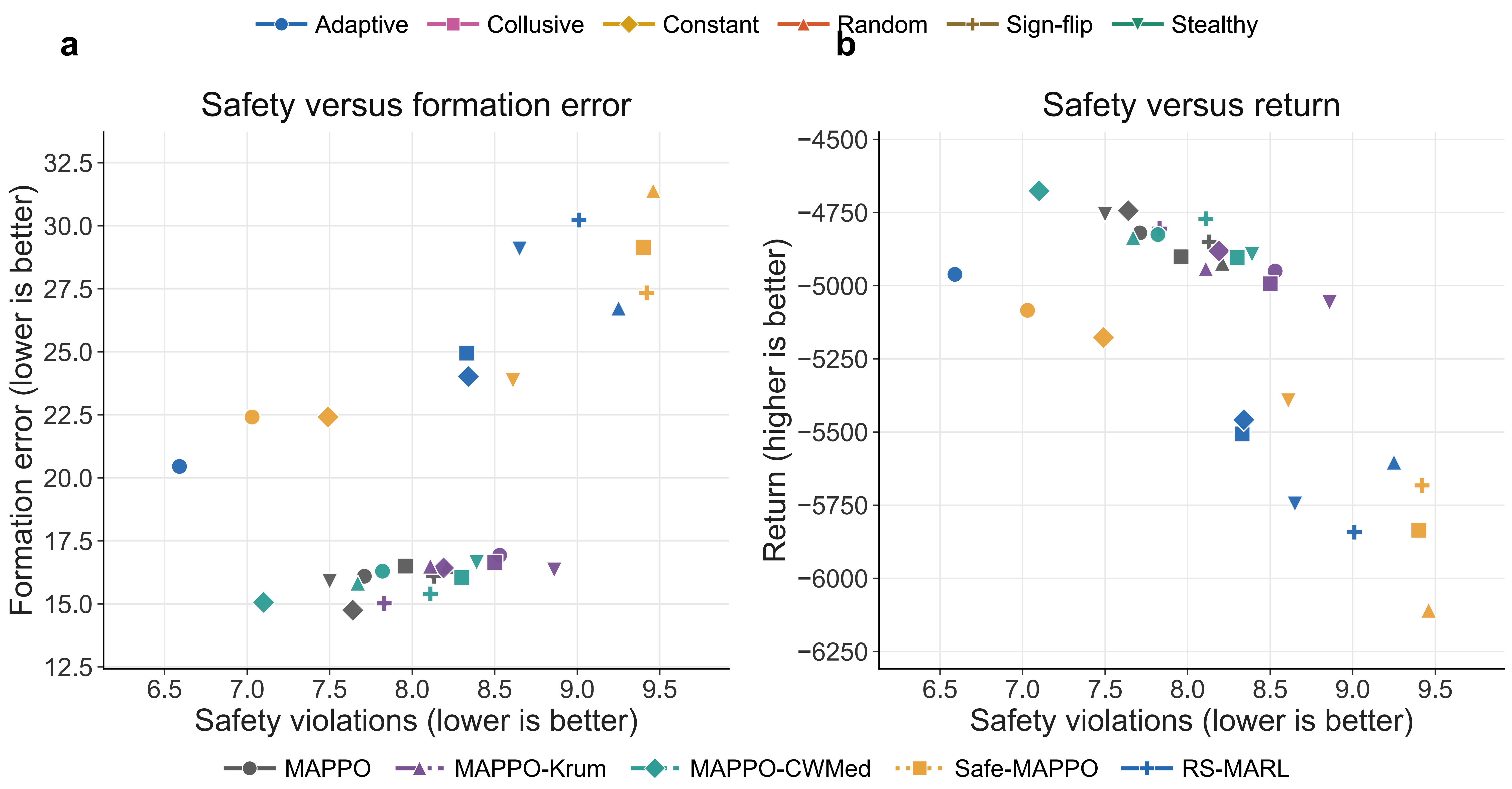

Adaptive
Collusive
Constant
Random
Sign-flip
Stealthy
a
b
Safety versus formation error
Formation error (lower is better)
Safety violations (lower is better)
Safety versus return
Return (higher is better)
Safety violations (lower is better)
MAPPO
MAPPO-Krum
MAPPO-CWMed
Safe-MAPPO
RS-MARL
