# Supplementary material for: Operating-Regime Evaluation of Byzantine-Resilient Multi-Agent Reinforcement Learning for Sensor-Networked Safe Formation Control
Source: Sensors (Basel). 2026 Jul 11;26(14):4408. doi: 10.3390/s26144408 (PMC13418820; doi:10.3390/s26144408)
Supplement: Supplementary file 1 [file sensors-26-04408-s001.zip › File_S1/figures/core/fig08_return_trends_journal.pdf]

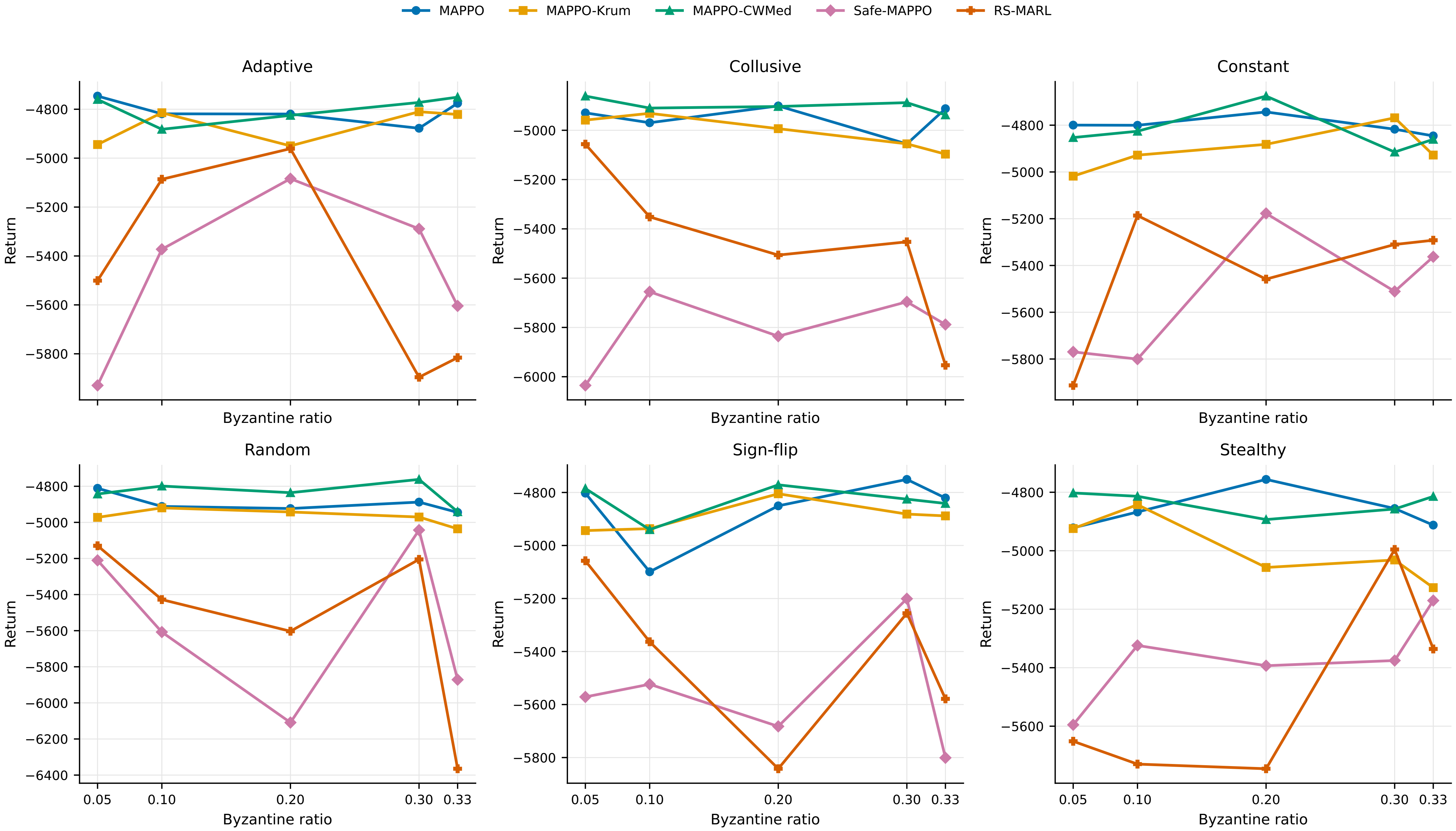

MAPPO
MAPPO-Krum
MAPPO-CWMed
Safe-MAPPO
RS-MARL
Adaptive
Collusive
Constant
Random
Sign-flip
Stealthy
Return
Byzantine ratio
0.05
0.10
0.20
0.30
0.33
−4800
−5000
−5200
−5400
−5600
−5800
−6000
−6200
−6400
