# Supplementary material for: Operating-Regime Evaluation of Byzantine-Resilient Multi-Agent Reinforcement Learning for Sensor-Networked Safe Formation Control
Source: Sensors (Basel). 2026 Jul 11;26(14):4408. doi: 10.3390/s26144408 (PMC13418820; doi:10.3390/s26144408)
Supplement: Supplementary file 1 [file sensors-26-04408-s001.zip › File_S1/figures/core/fig_scope_heatmap.pdf]

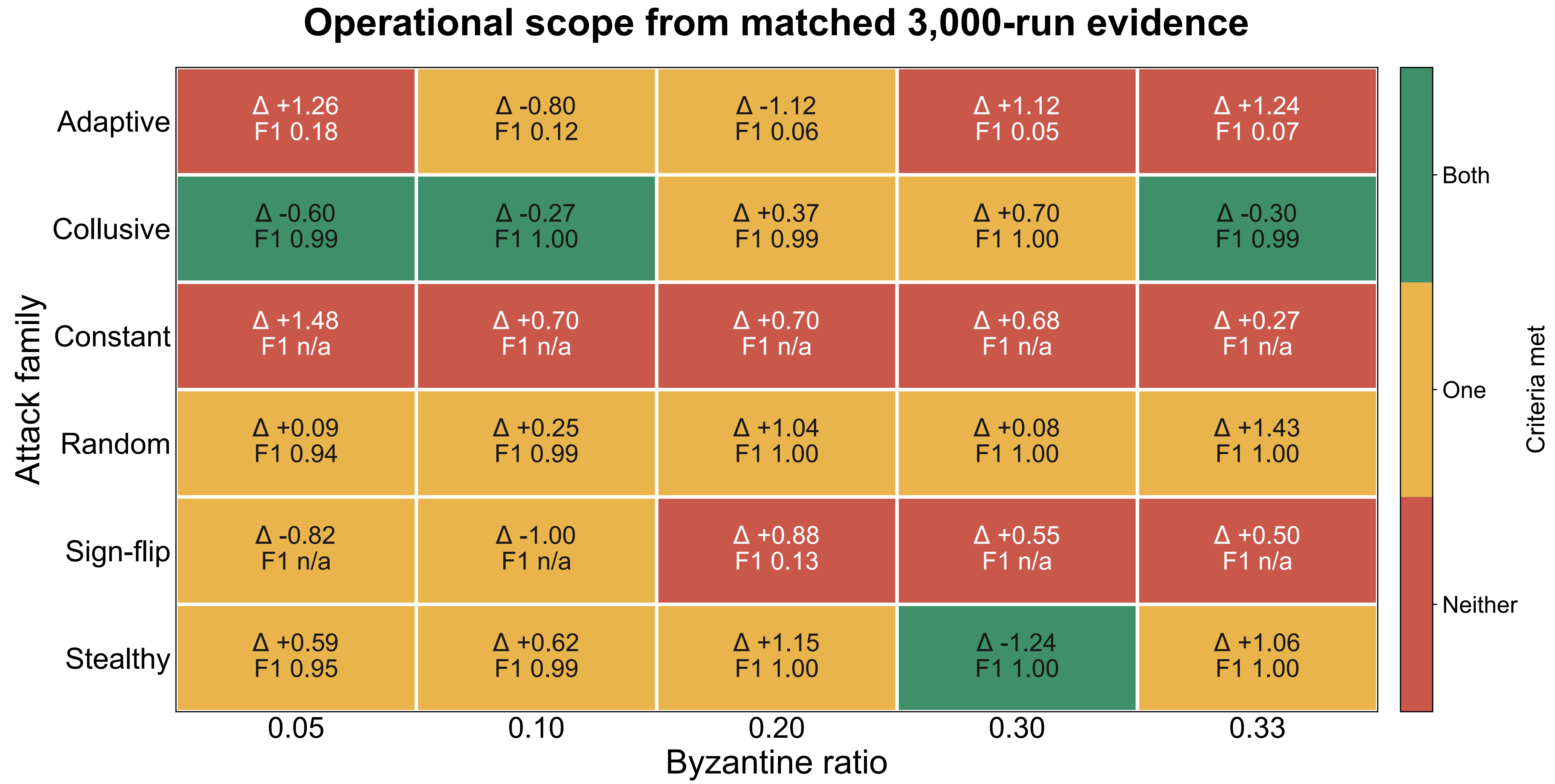

Detection-capable: RS-MARL F1 > 0.5. Safety-improving: fewer mean violations than MAPPO.
Cell labels show safety delta (RS-MARL - MAPPO; negative is better) and detection F1.
