# Supplementary figures and images for: Operating-Regime Evaluation of Byzantine-Resilient Multi-Agent Reinforcement Learning for Sensor-Networked Safe Formation Control
Source: Sensors (Basel). 2026 Jul 11;26(14):4408. doi: 10.3390/s26144408 (PMC13418820; doi:10.3390/s26144408)

## Ablation: Detection F1

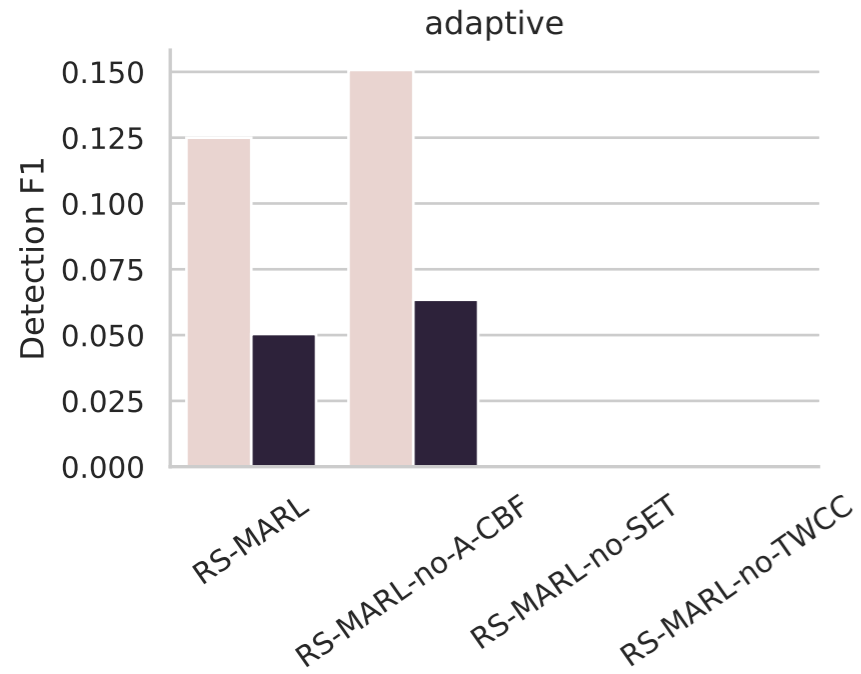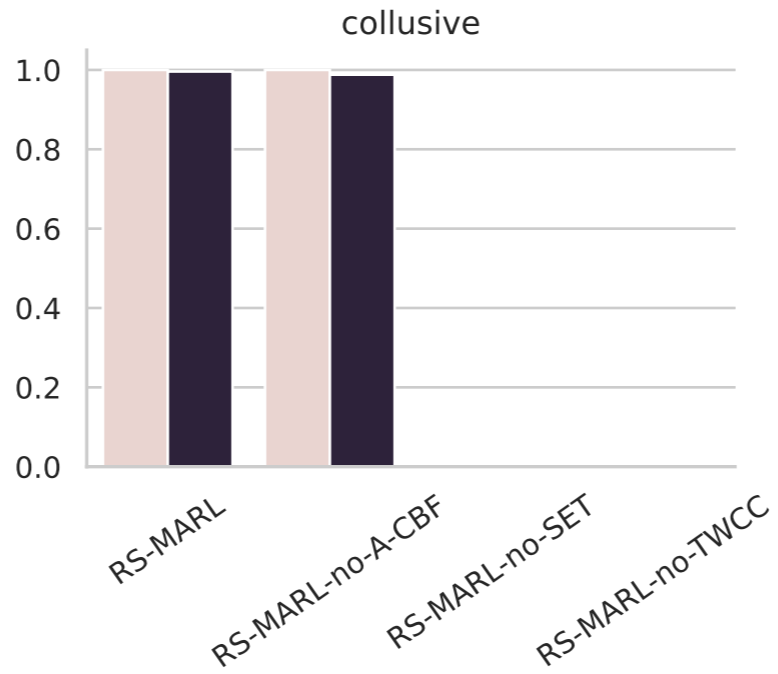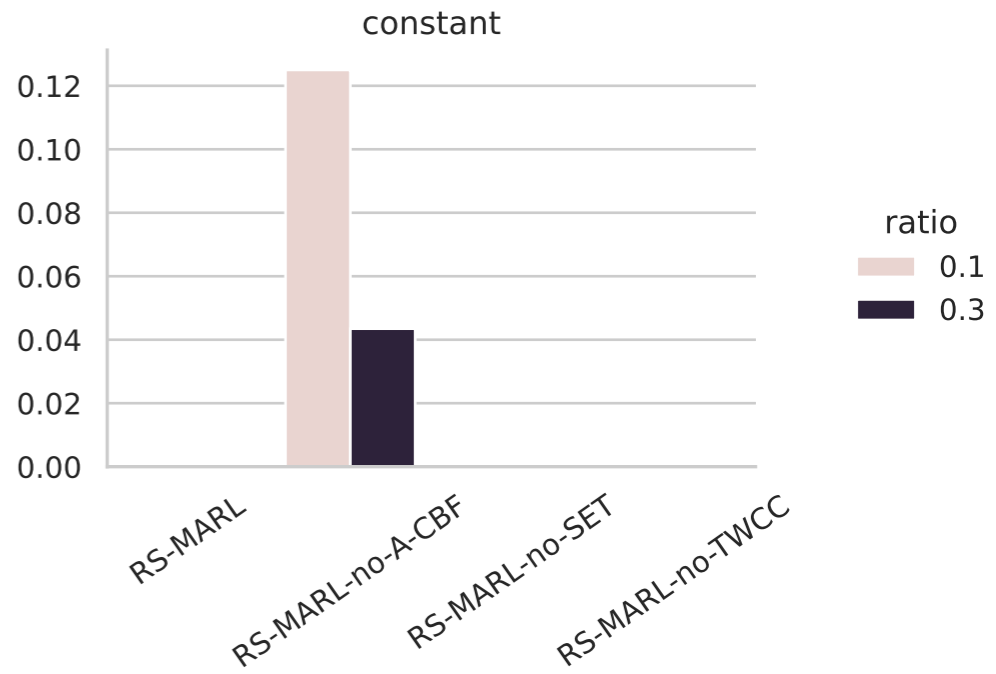

Supplement: Supplementary file 1 [file sensors-26-04408-s001.zip › File_S1/figures/canonical_580/ablation_final_f1_mean.pdf]

## Ablation: Formation Error

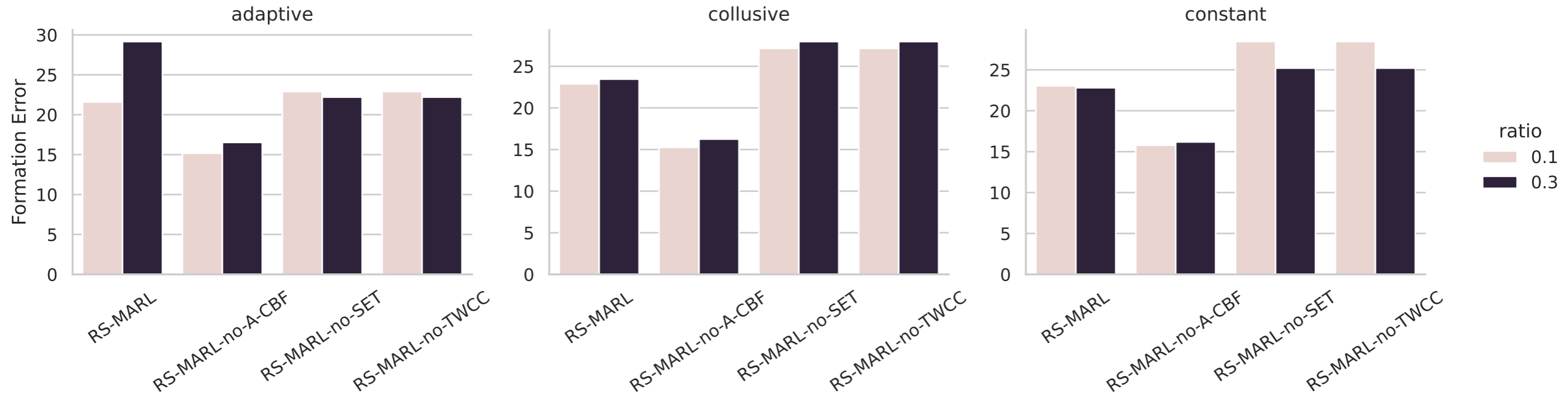

Supplement: Supplementary file 1 [file sensors-26-04408-s001.zip › File_S1/figures/canonical_580/ablation_final_fe_mean.pdf]

Ablation: Return

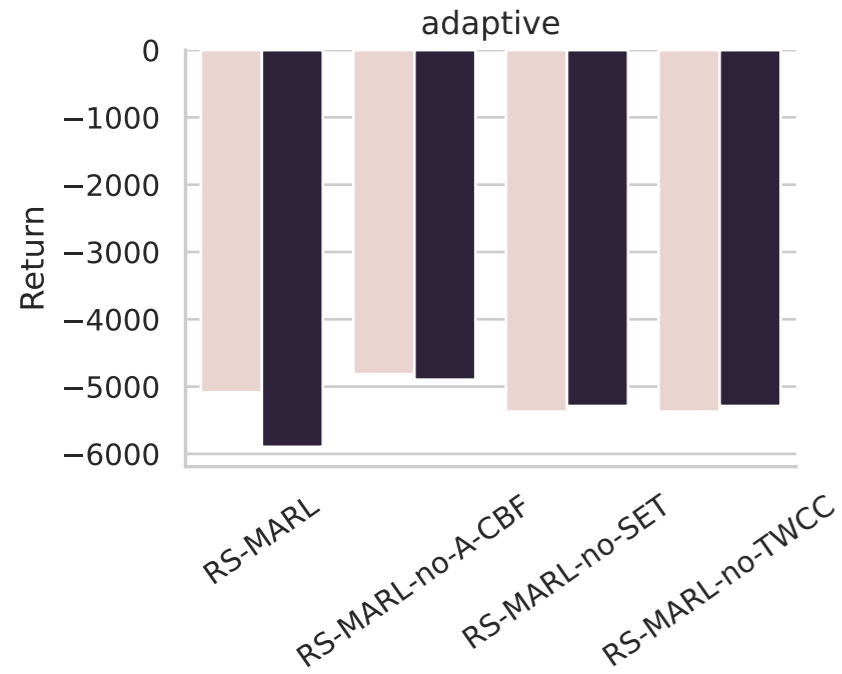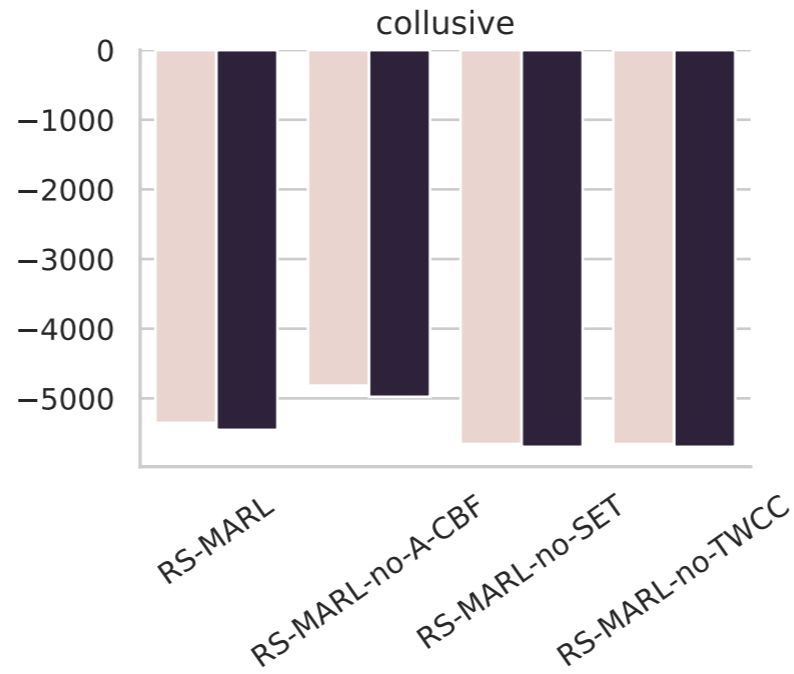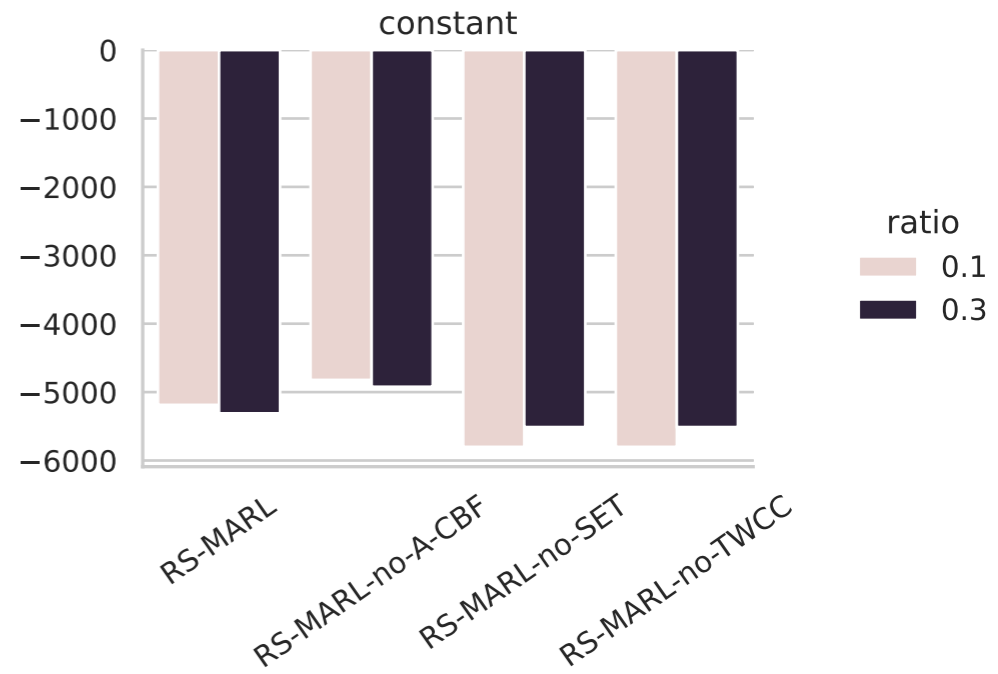

Supplement: Supplementary file 1 [file sensors-26-04408-s001.zip › File_S1/figures/canonical_580/ablation_final_return_mean.pdf]

## Ablation: Safety Violations

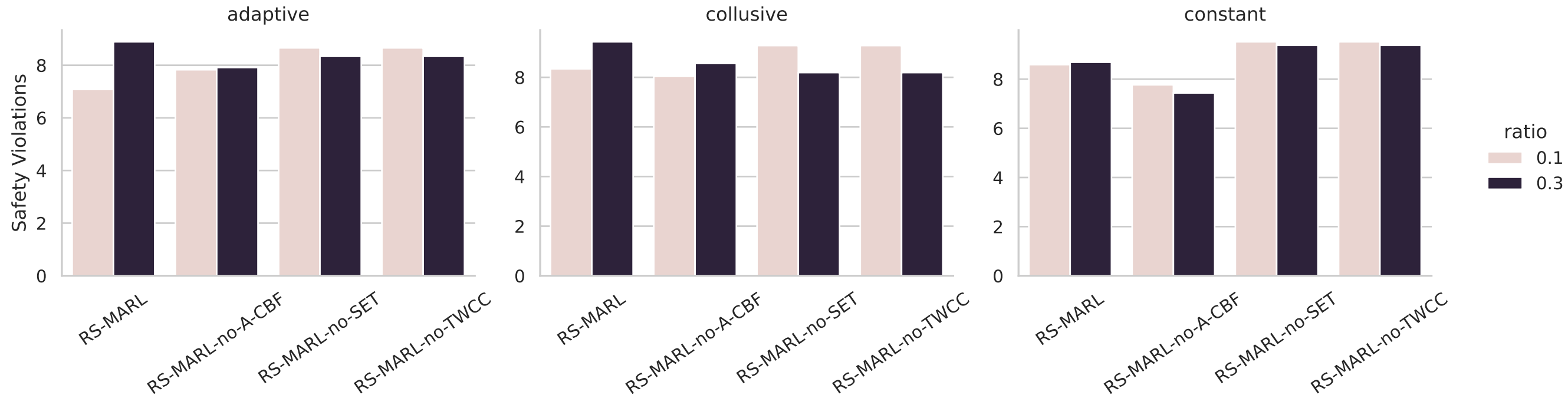

Supplement: Supplementary file 1 [file sensors-26-04408-s001.zip › File_S1/figures/canonical_580/ablation_final_safety_violations_mean.pdf]

Benign: Detection F1

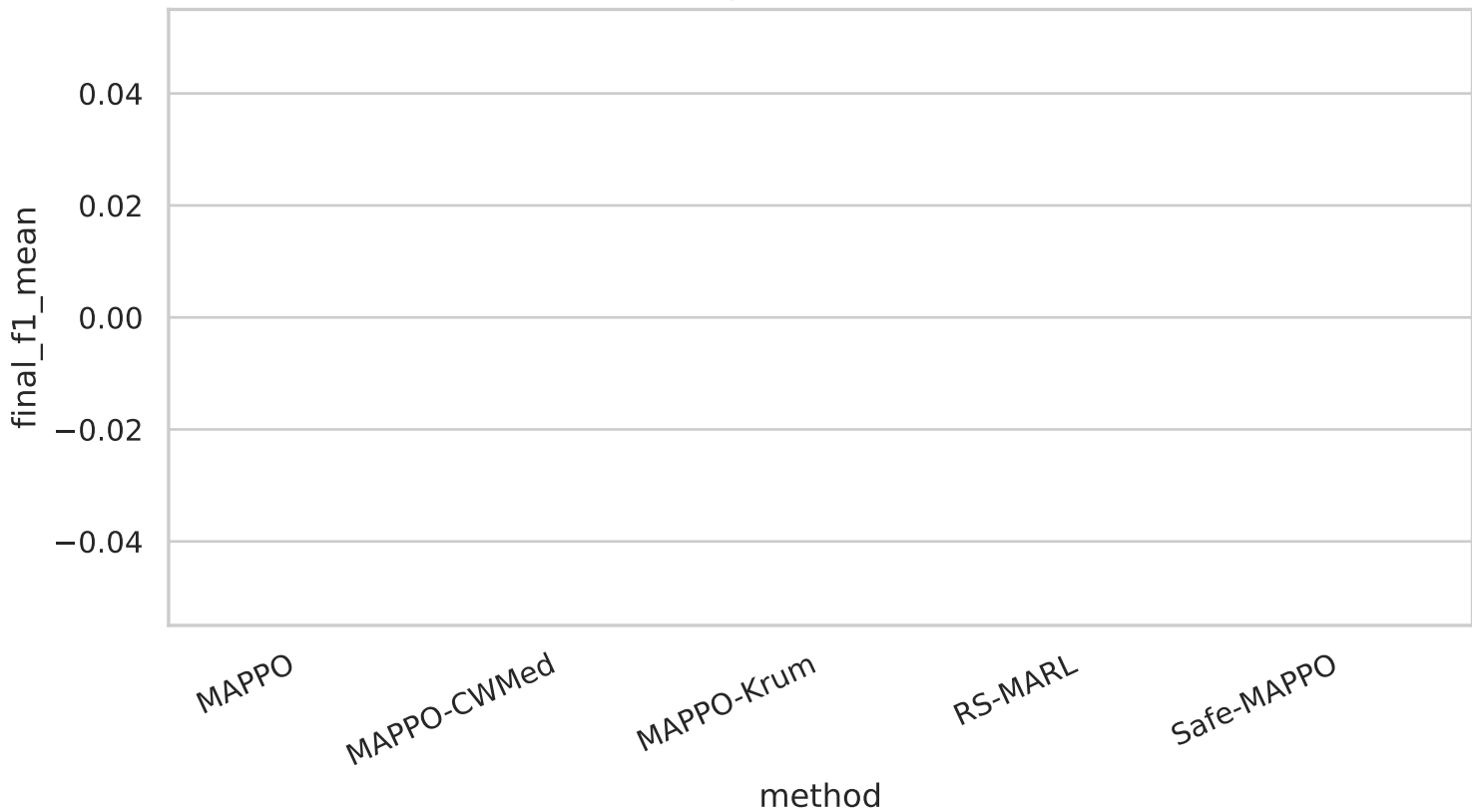

Supplement: Supplementary file 1 [file sensors-26-04408-s001.zip › File_S1/figures/canonical_580/benign_final_f1_mean.pdf]

Benign: Formation Error

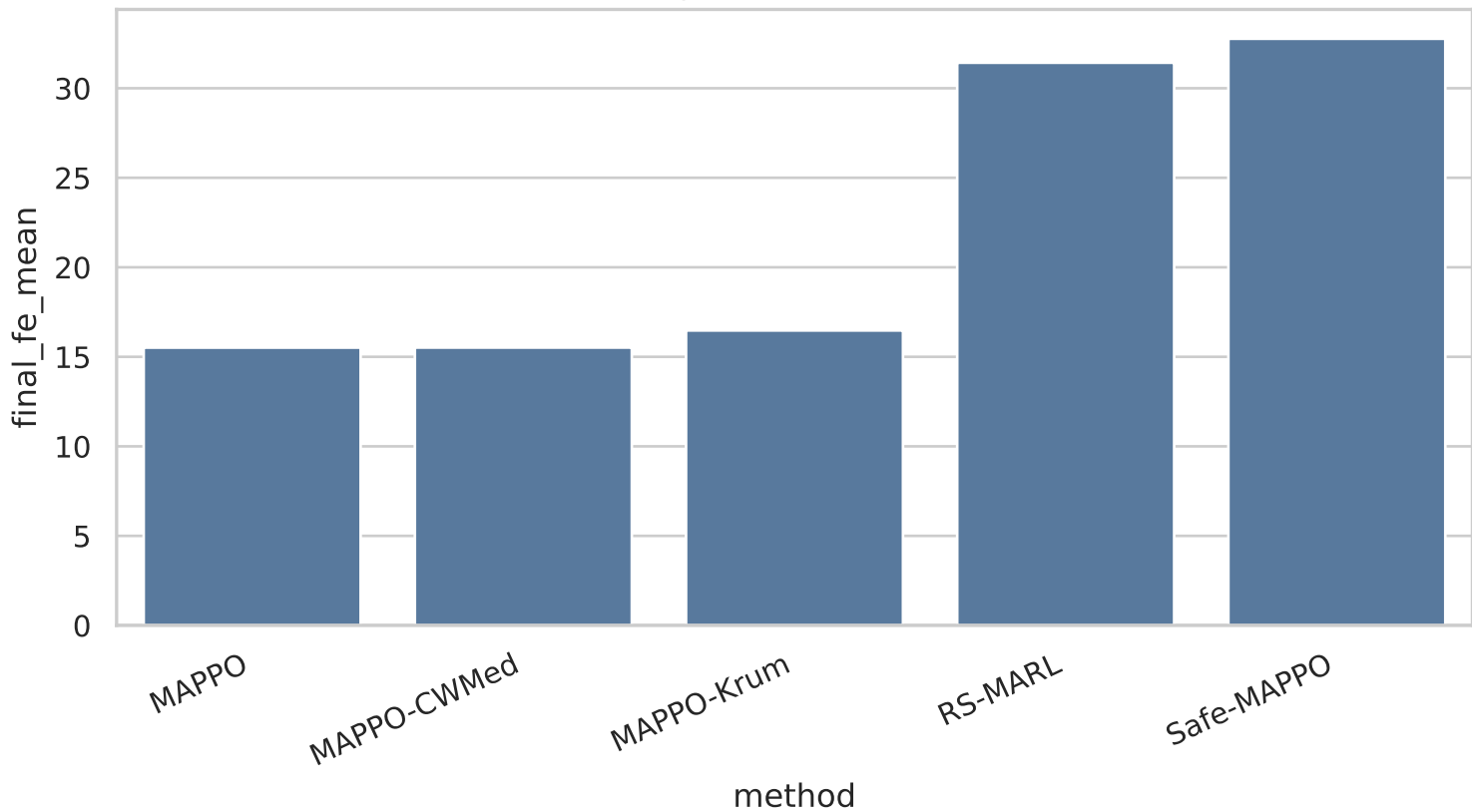

Supplement: Supplementary file 1 [file sensors-26-04408-s001.zip › File_S1/figures/canonical_580/benign_final_fe_mean.pdf]

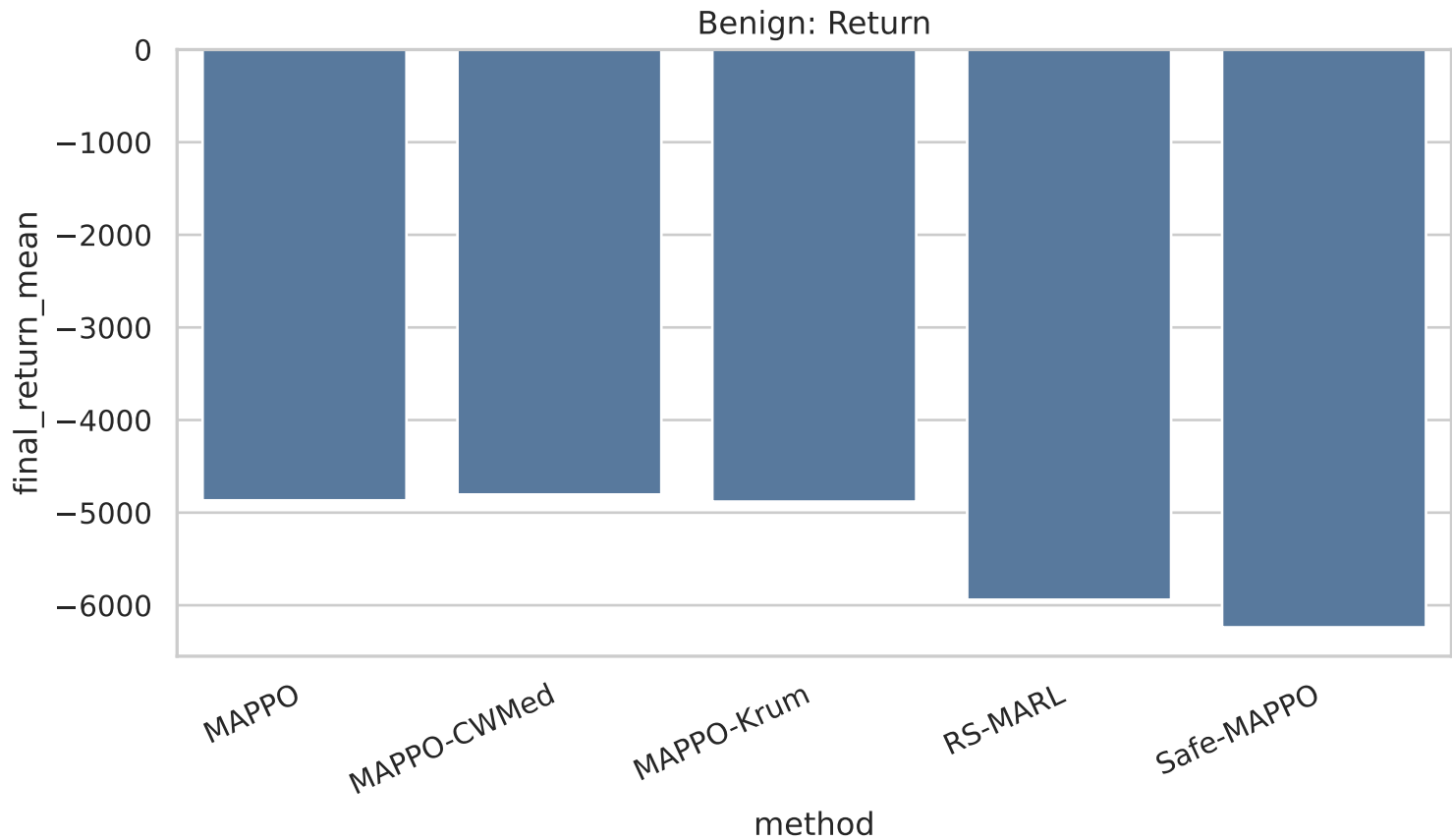

Supplement: Supplementary file 1 [file sensors-26-04408-s001.zip › File_S1/figures/canonical_580/benign_final_return_mean.pdf]

Benign: Safety Violations

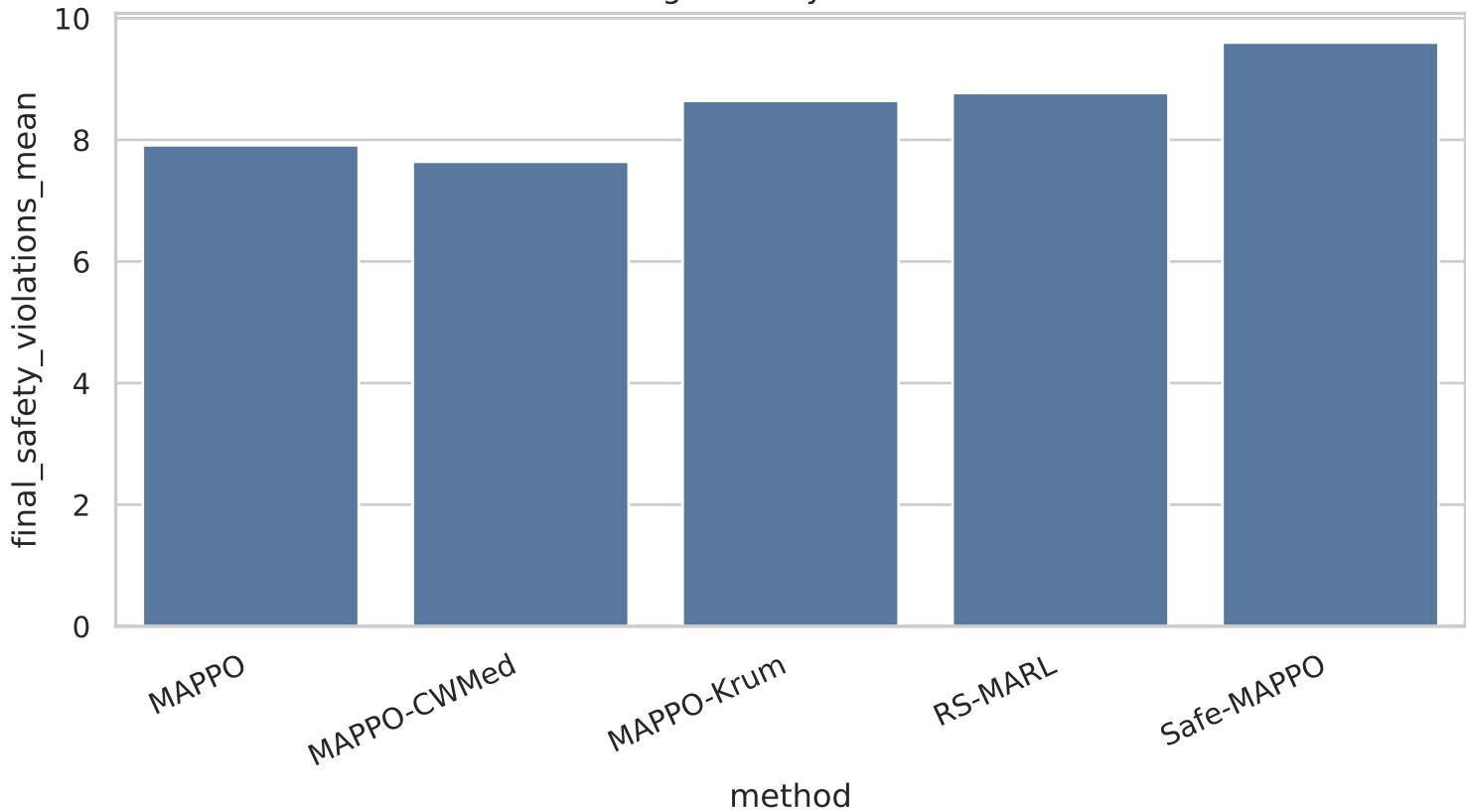

Supplement: Supplementary file 1 [file sensors-26-04408-s001.zip › File_S1/figures/canonical_580/benign_final_safety_violations_mean.pdf]

MAPPO RS-MARL

Adaptive

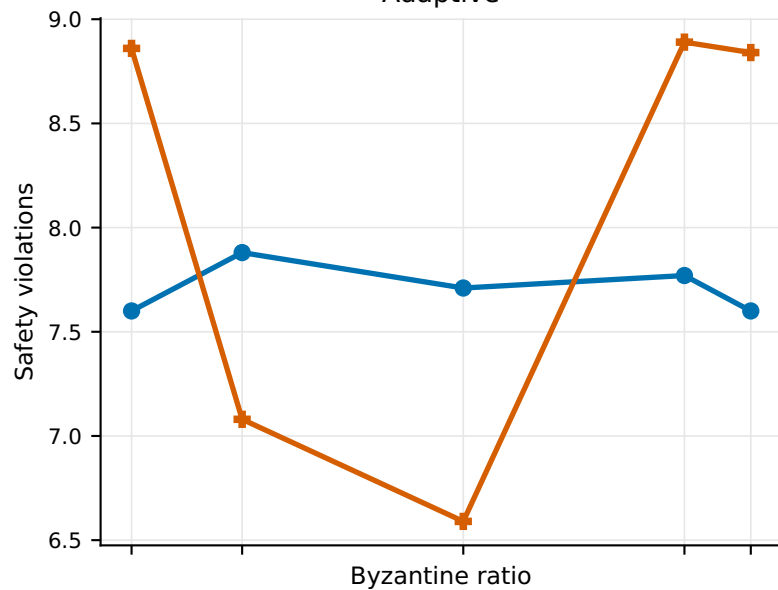

Collusive

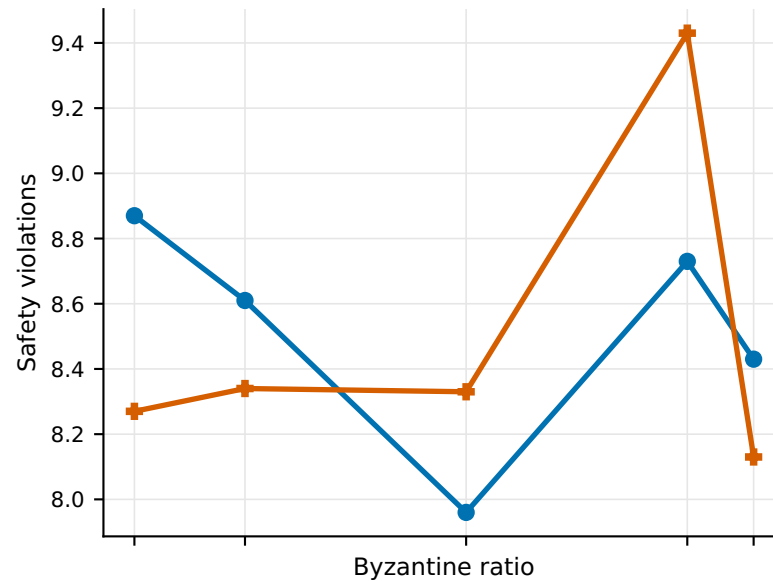

Constant

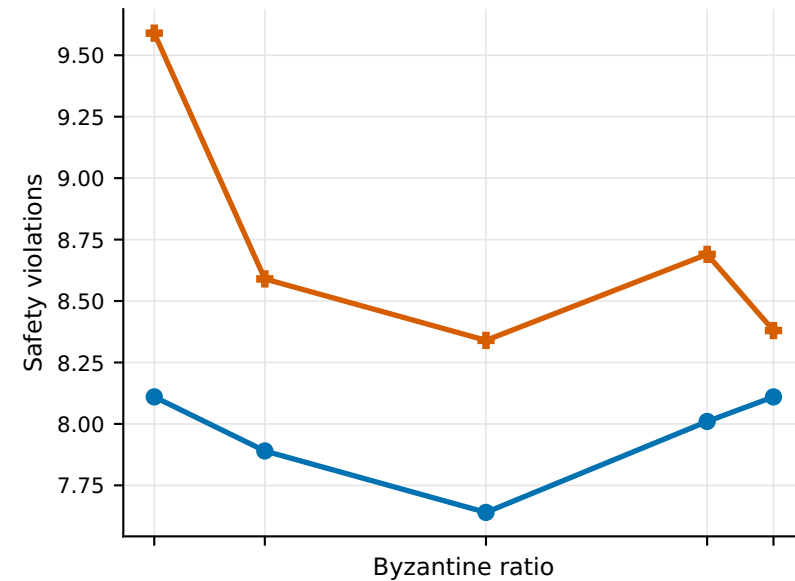

Random

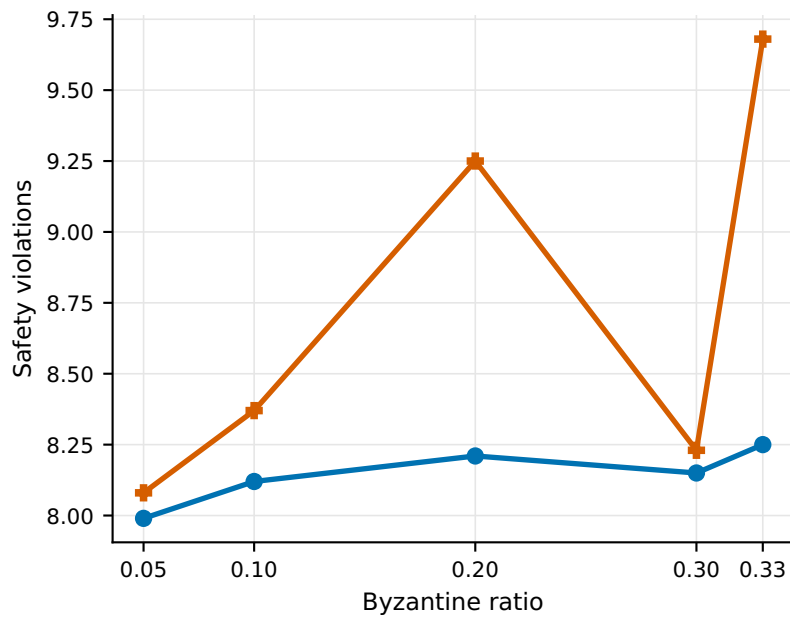

Sign-flip

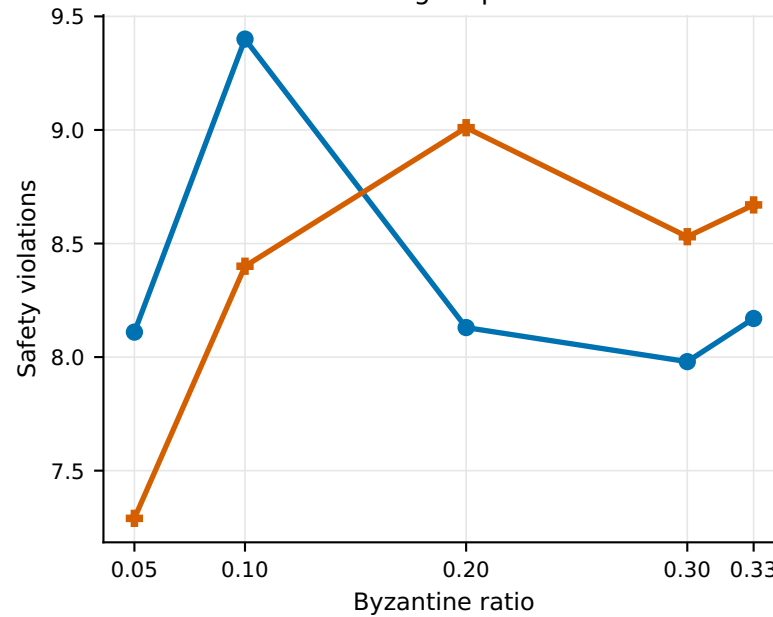

Stealthy

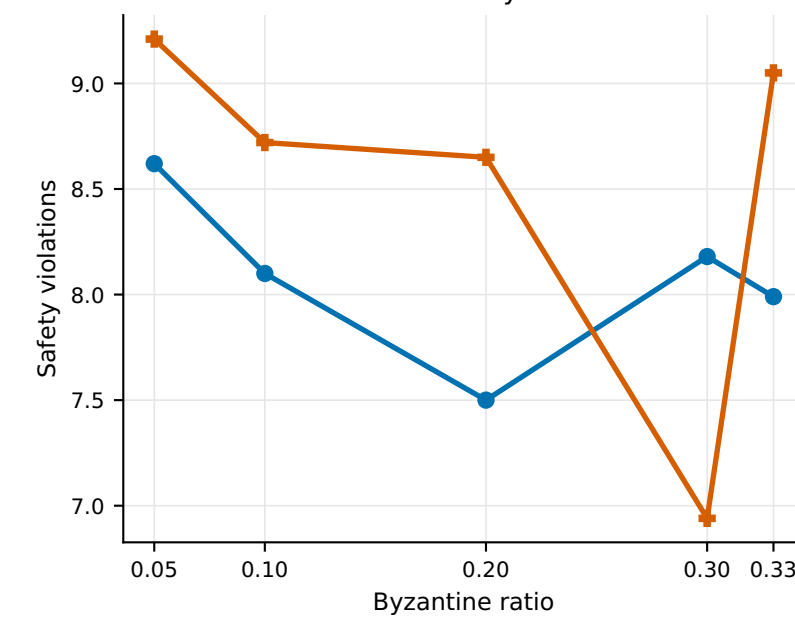

Supplement: Supplementary file 1 [file sensors-26-04408-s001.zip › File_S1/figures/core/curve_all_attacks_safety_mappo_vs_rsmarl.pdf]

# Representative safety gains under Byzantine attacks

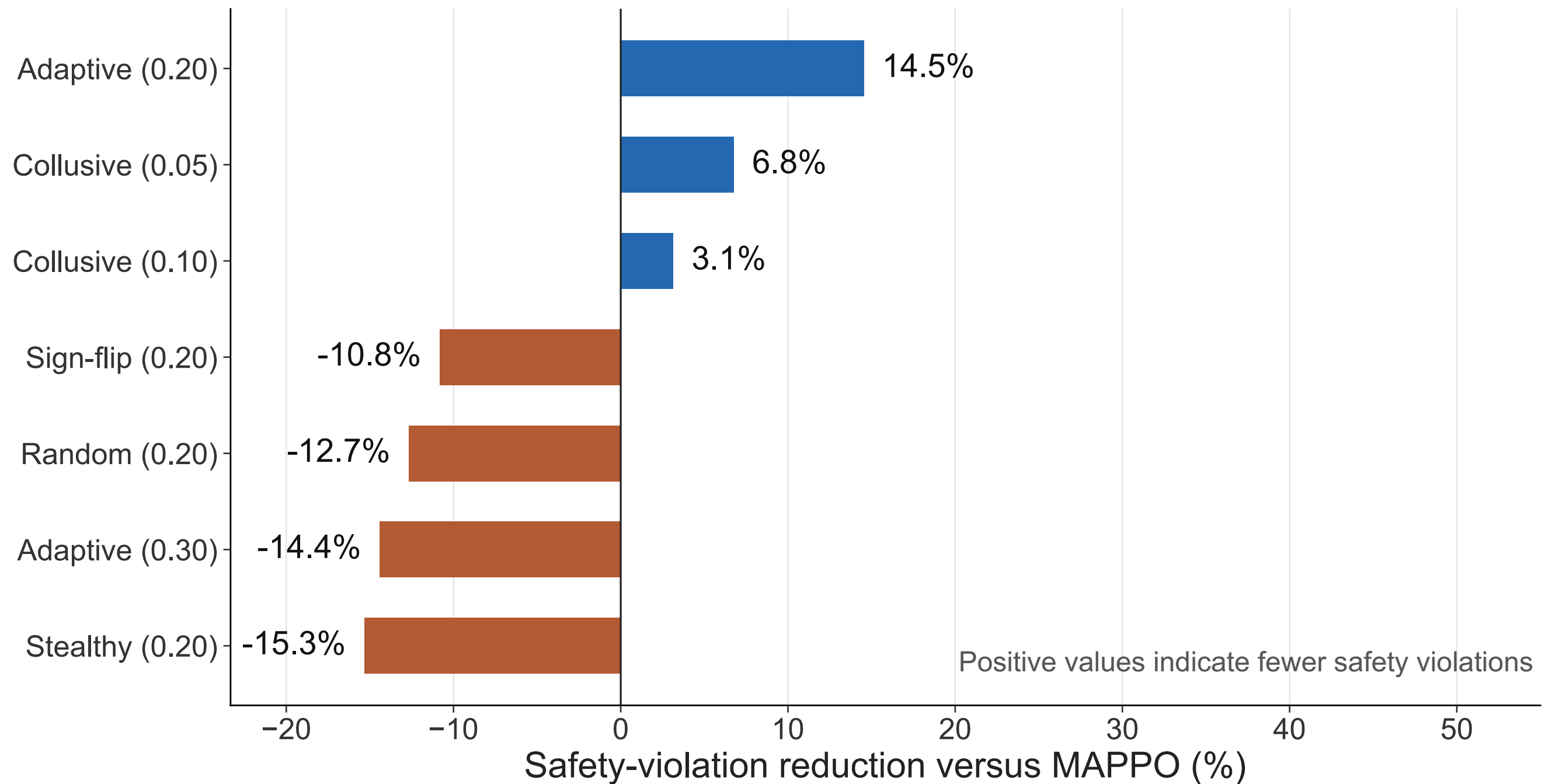

Supplement: Supplementary file 1 [file sensors-26-04408-s001.zip › File_S1/figures/core/fig01_safety_reduction_horizontal.pdf]

Safety-performance trade-off at 20% Byzantine agents

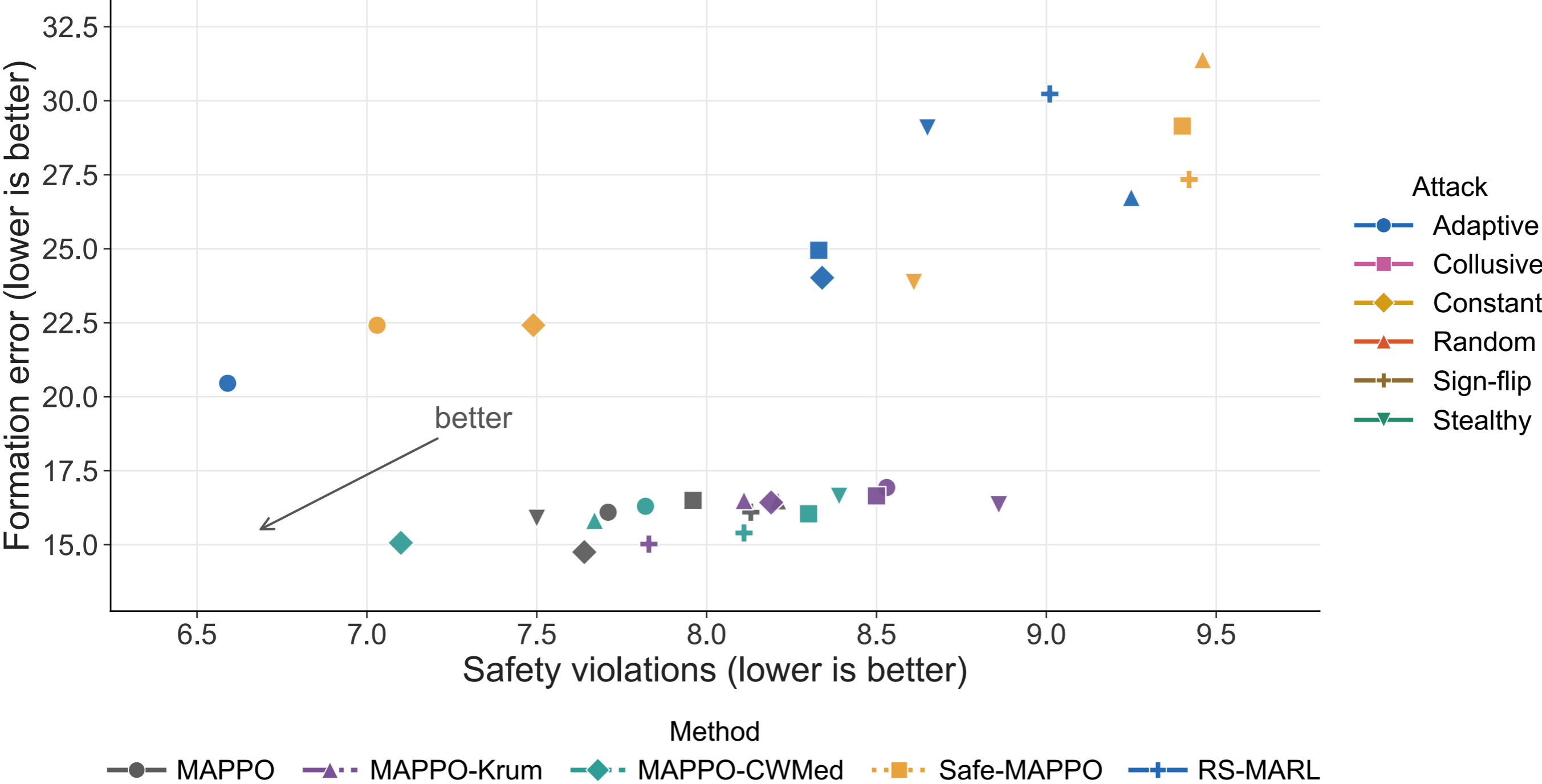

Supplement: Supplementary file 1 [file sensors-26-04408-s001.zip › File_S1/figures/core/fig02_tradeoff_scatter.pdf]

Trust-estimator F1 across Byzantine ratios

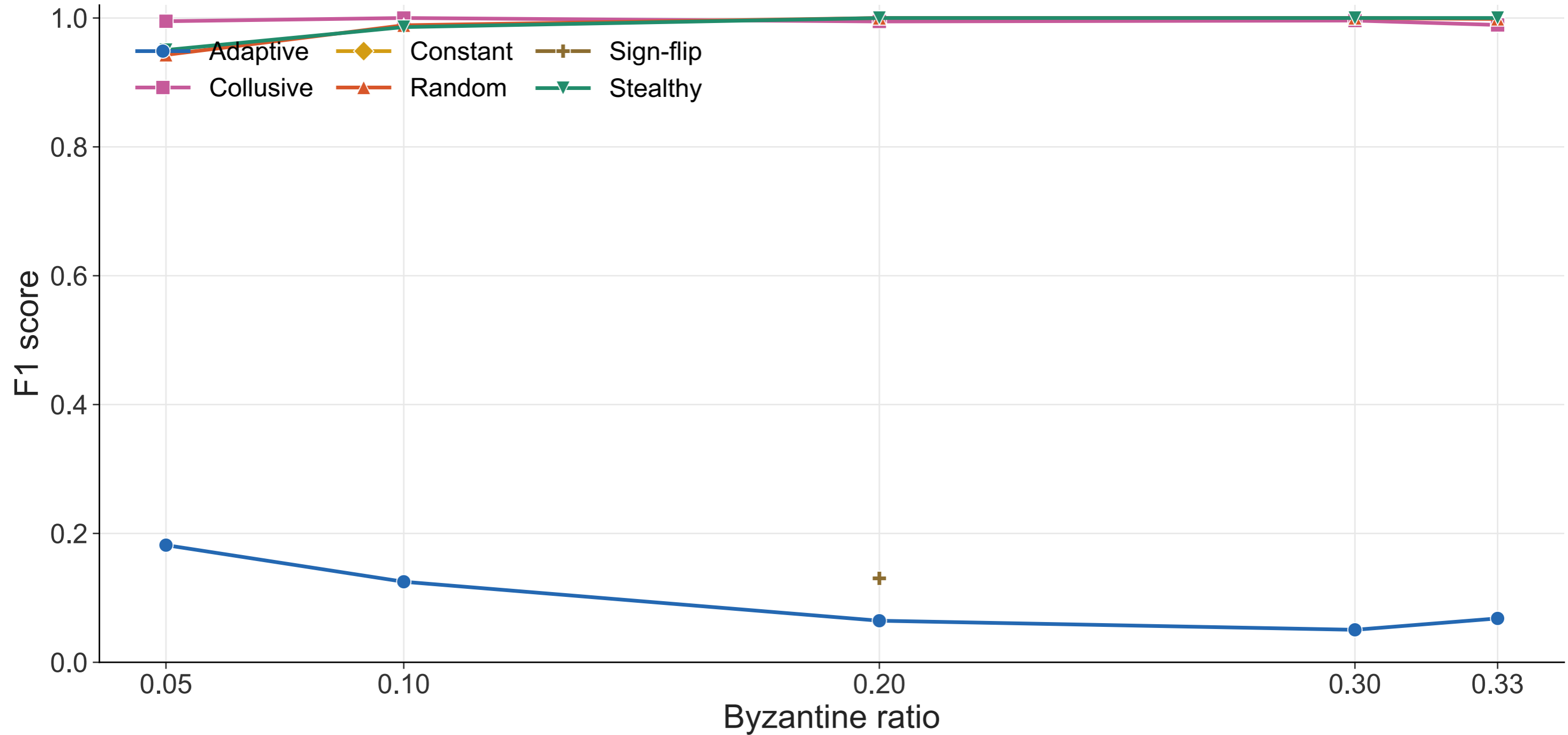

Supplement: Supplementary file 1 [file sensors-26-04408-s001.zip › File_S1/figures/core/fig03_detection_f1.pdf]

**a**

Safety-violation reduction

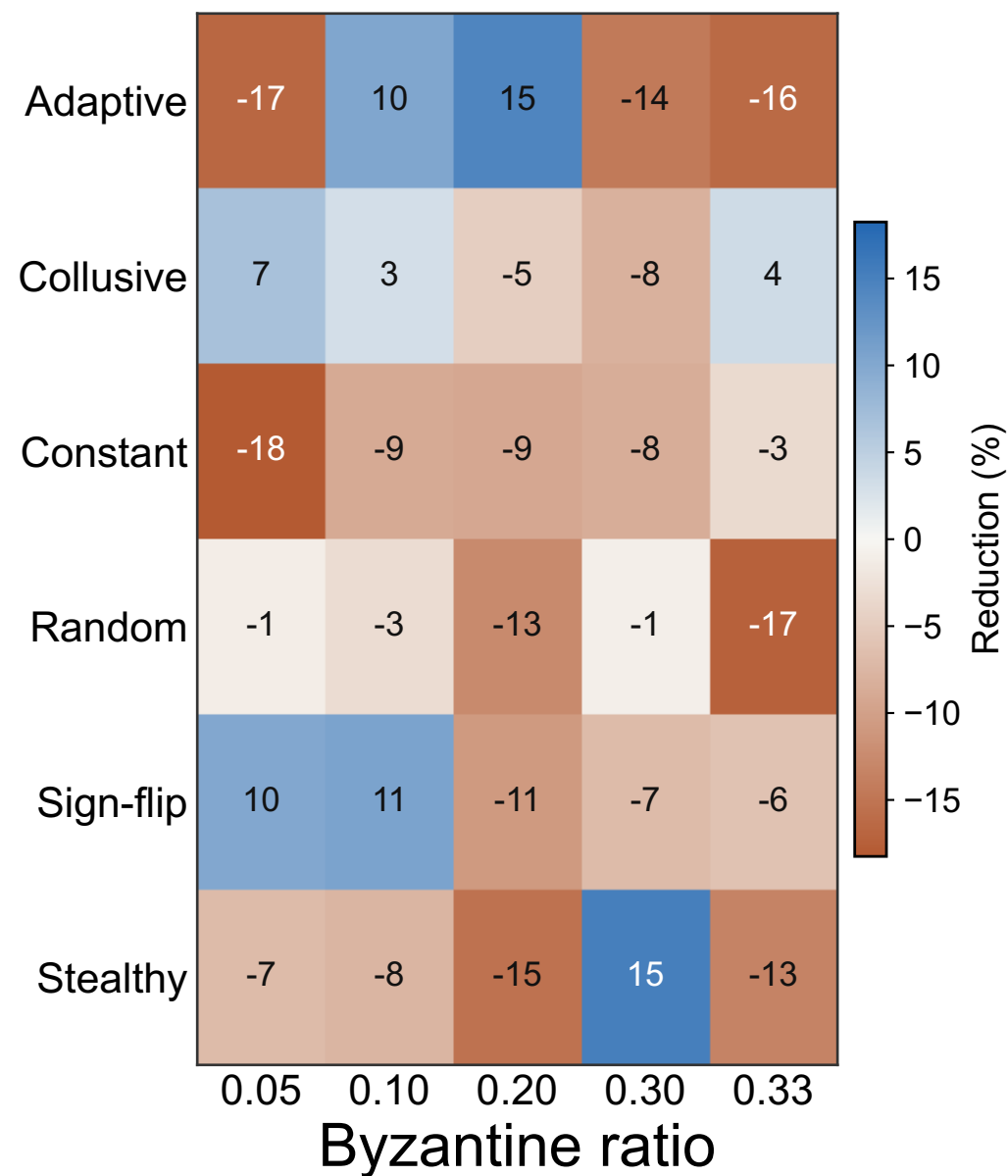**b**

Formation-error change

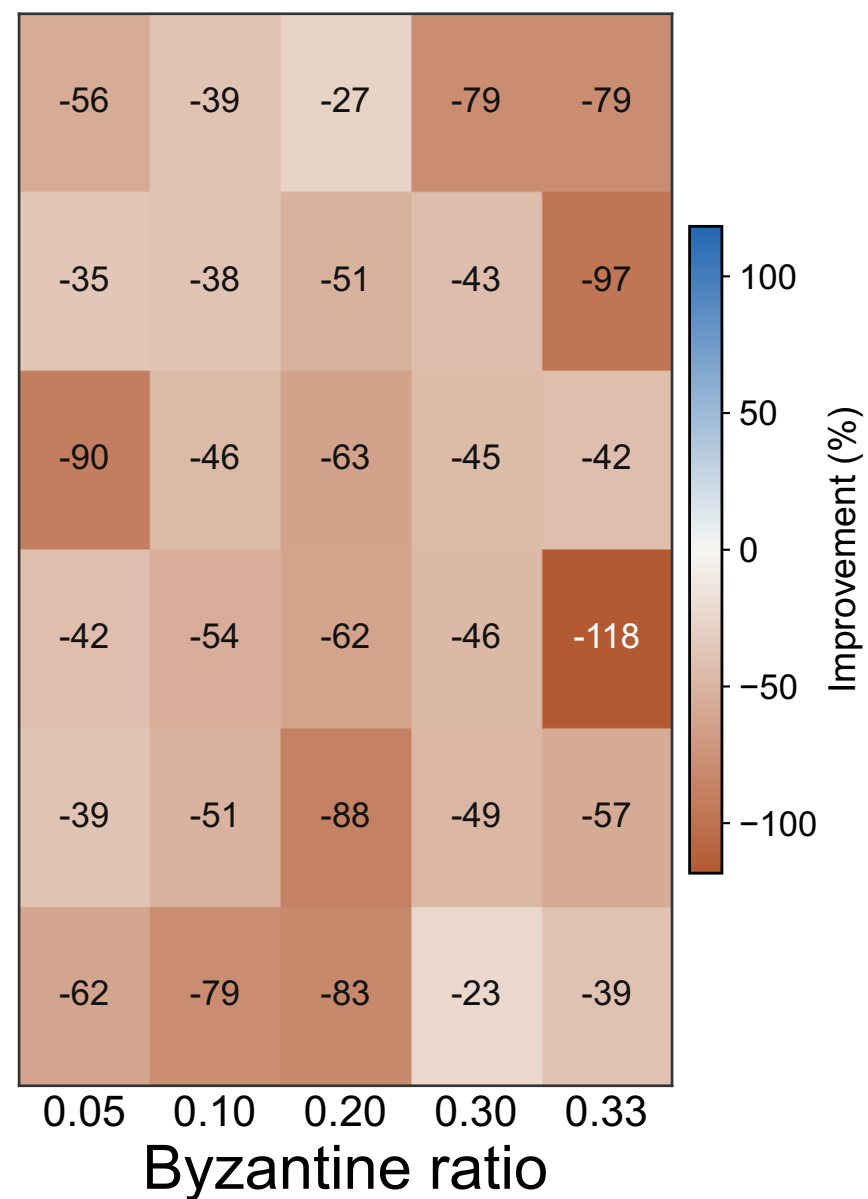**c**

Return difference

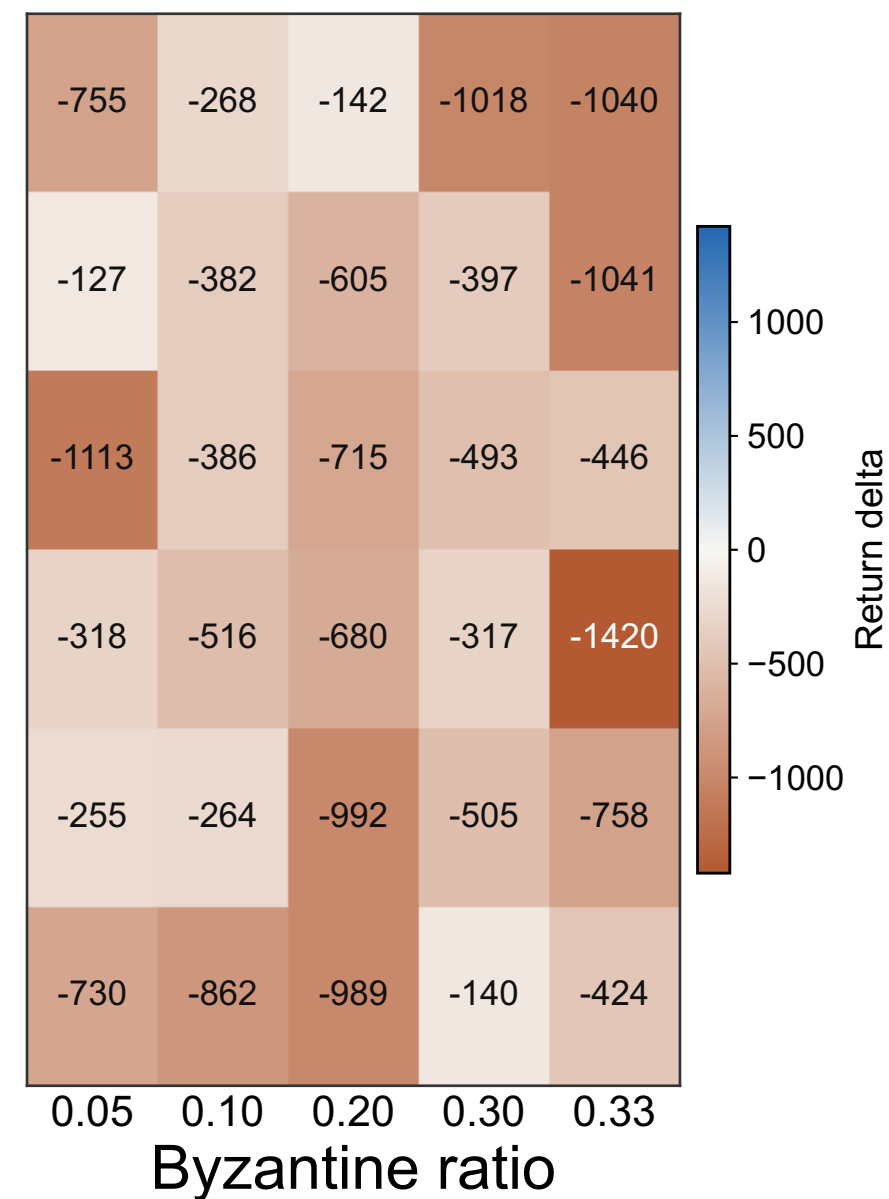

Supplement: Supplementary file 1 [file sensors-26-04408-s001.zip › File_S1/figures/core/fig07_delta_heatmaps_journal.pdf]

Adaptive Collusive Constant Random Sign-flip Stealthy

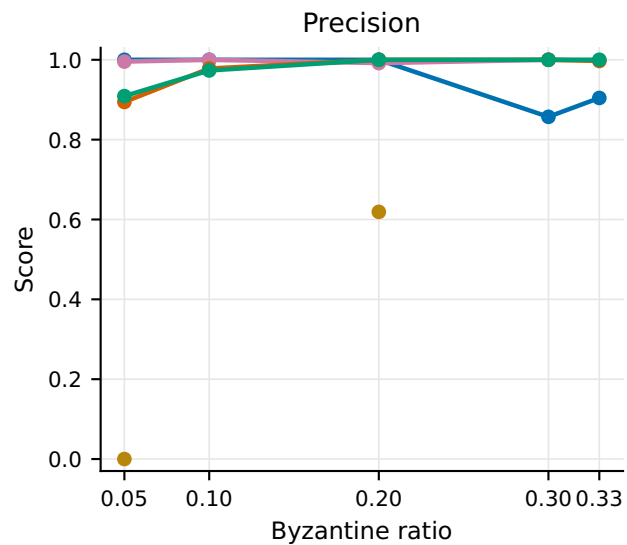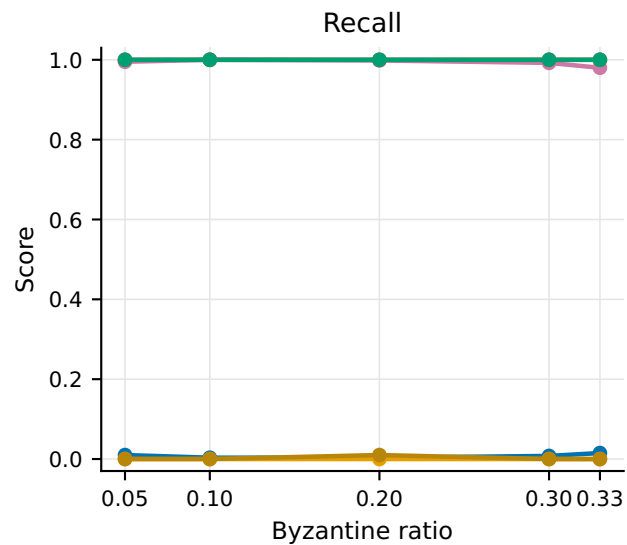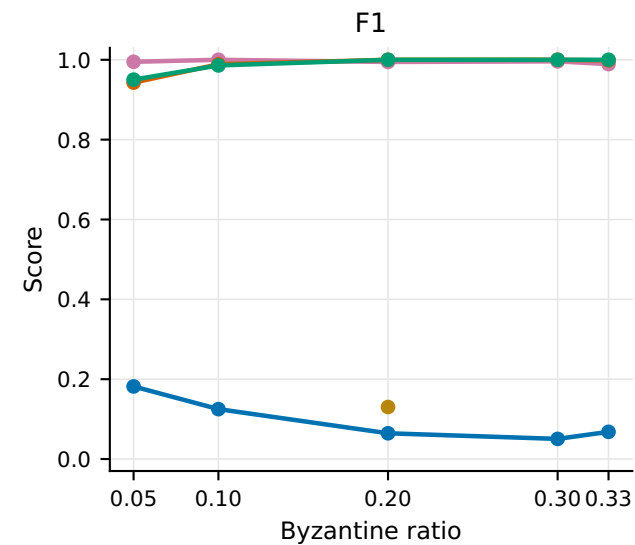

RS-MARL trust-estimator F1

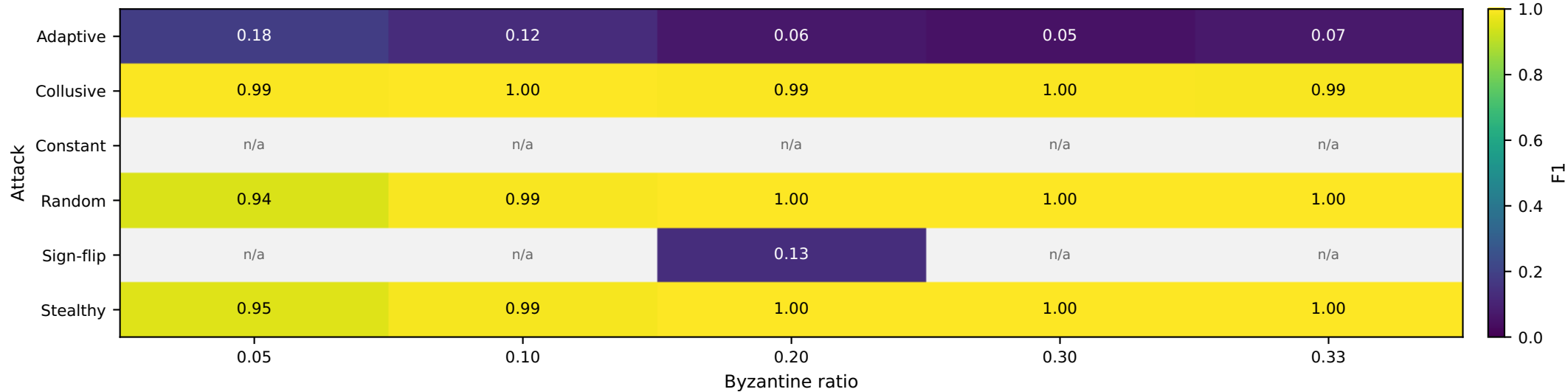

Supplement: Supplementary file 1 [file sensors-26-04408-s001.zip › File_S1/figures/core/fig10_detection_journal.pdf]

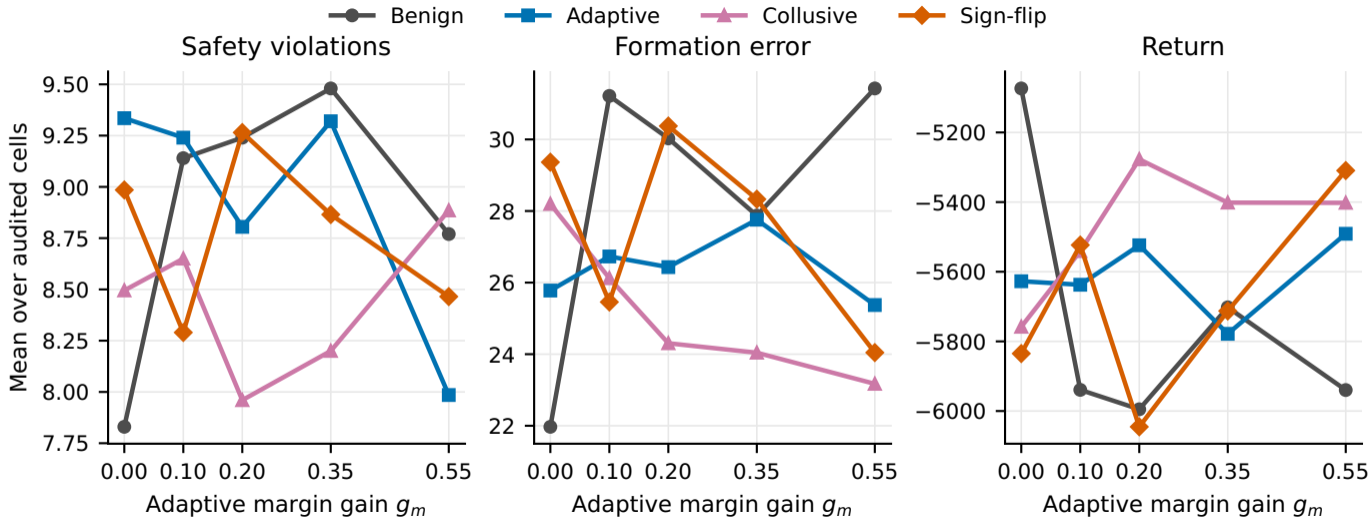

Supplement: Supplementary file 1 [file sensors-26-04408-s001.zip › File_S1/figures/review_audit_v2/fig_review_v2_acbf_calibration.pdf]

Safety violations

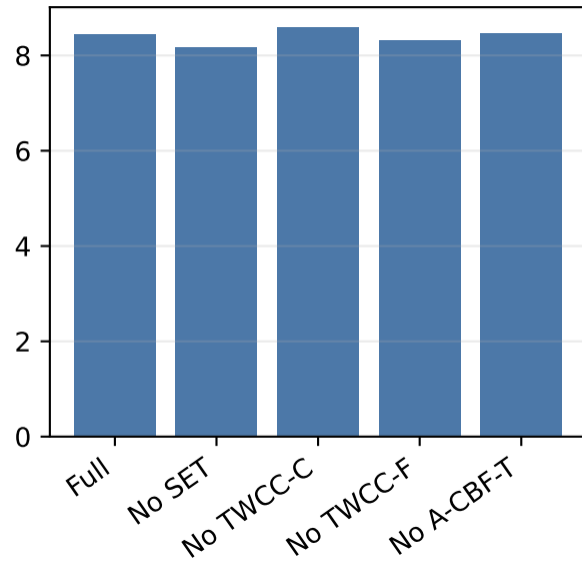

Formation error

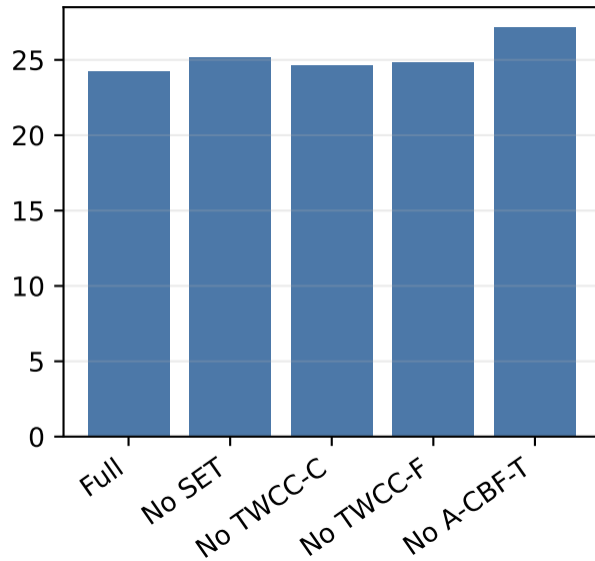

Return

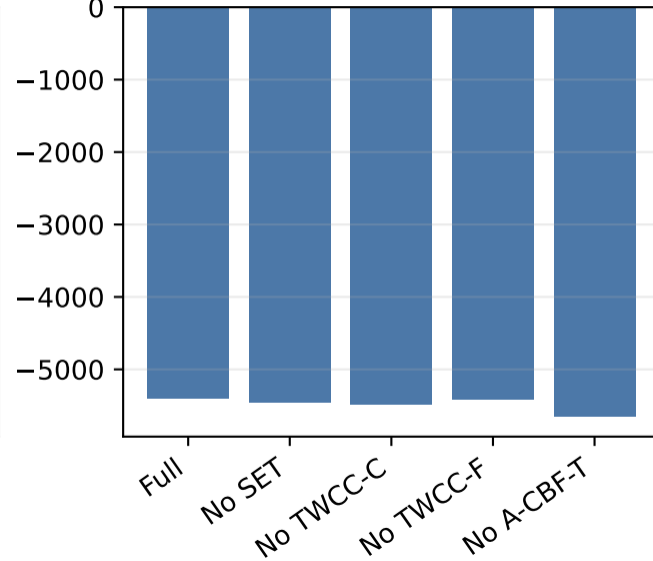

Detection F1

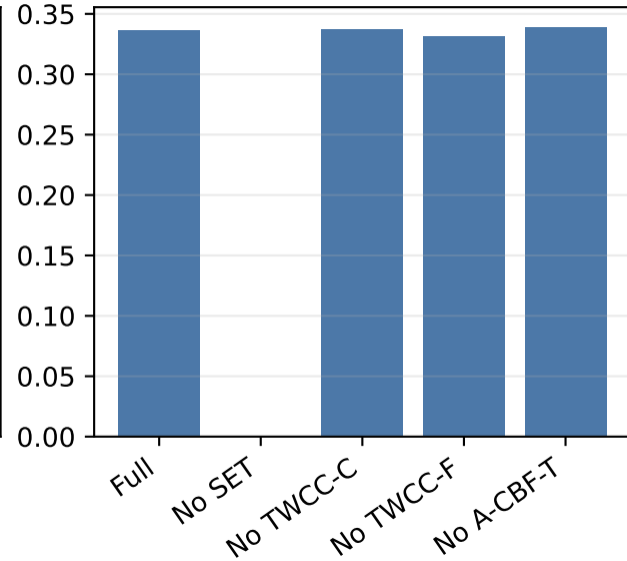

Supplement: Supplementary file 1 [file sensors-26-04408-s001.zip › File_S1/figures/review_audit_v2/fig_review_v2_four_switch_ablation.pdf]

# Review-audit V2 n=100 key-domain confirmation

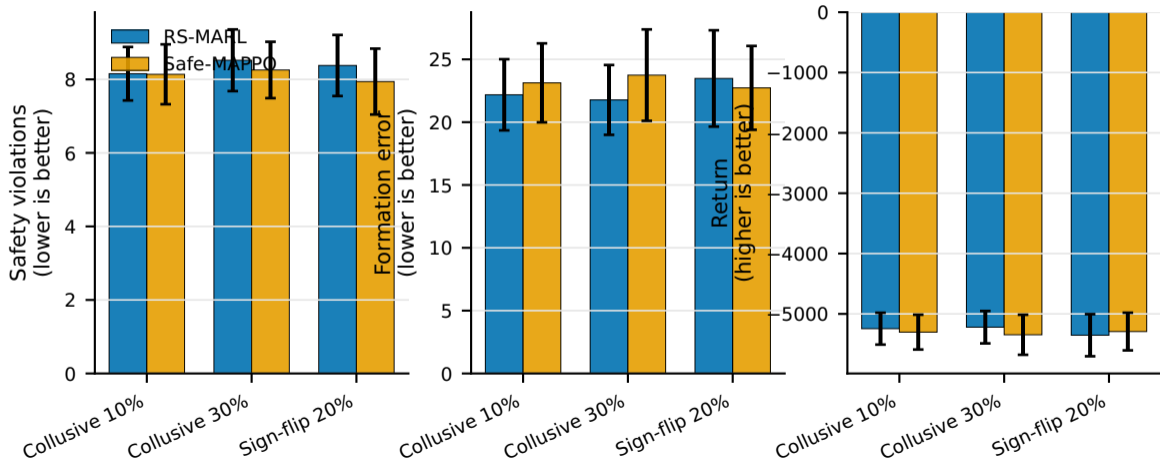

Supplement: Supplementary file 1 [file sensors-26-04408-s001.zip › File_S1/figures/review_audit_v2/fig_review_v2_key_domain_n100.pdf]

# Review-audit V2 sensor-impairment mini-grid

RS-MARL      Safe-MAPPO

Benign

Collusive, 30%

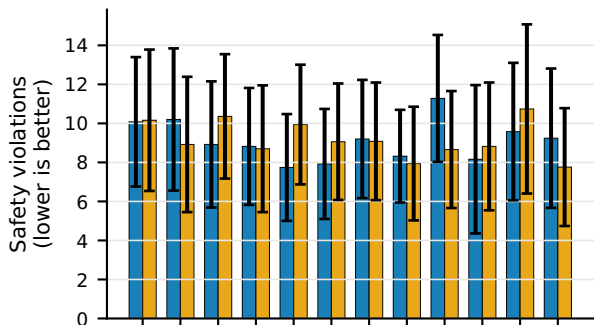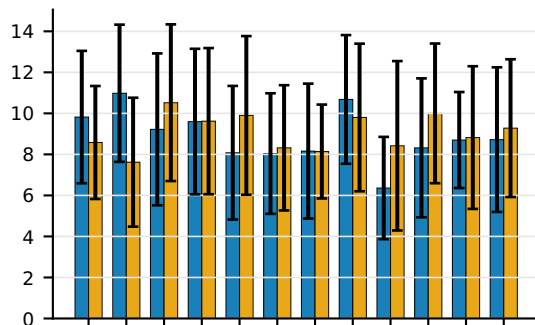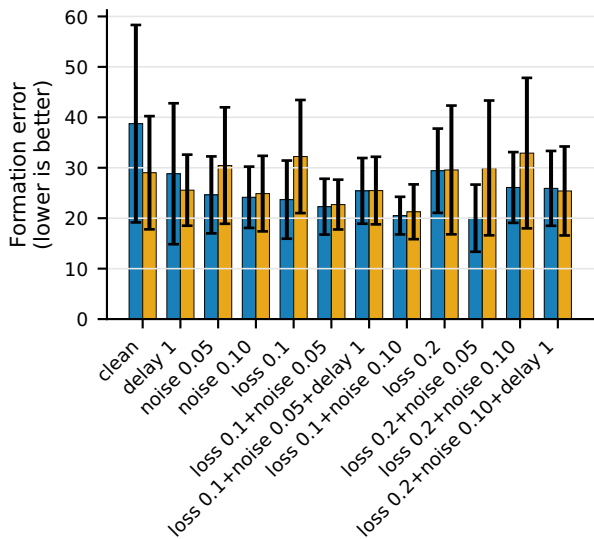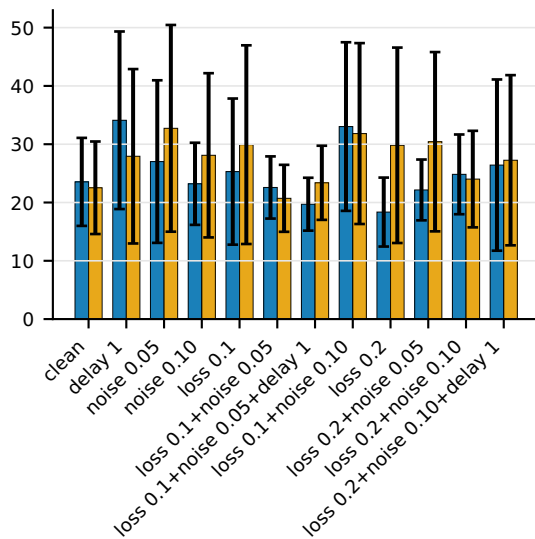

Supplement: Supplementary file 1 [file sensors-26-04408-s001.zip › File_S1/figures/review_audit_v2/fig_review_v2_sensor_impairment.pdf]
